# Diversity and evolution of prokaryotic viral lytic proteins

**DOI:** 10.1093/ismejo/wraf200

**Published:** 2025-10-08

**Authors:** Ting Yang, Mujie Zhang, Yi Yi, Yecheng Wang, Zhiwei Wang, Rui Zhang, Xiang Xiao, Huahua Jian

**Affiliations:** State Key Laboratory of Microbial Metabolism, Joint International Research Laboratory of Metabolic & Development Sciences, School of Life Sciences & Biotechnology, Shanghai Jiao Tong University, 800 Dongchuan Road, Minhang District, Shanghai 200240, PR China; Yazhou Bay Institute of Deepsea Sci-Tech, Shanghai Jiao Tong University, No. 2 Road, Yazhou District, Sanya 572025, Hainan, PR China; State Key Laboratory of Microbial Metabolism, Joint International Research Laboratory of Metabolic & Development Sciences, School of Life Sciences & Biotechnology, Shanghai Jiao Tong University, 800 Dongchuan Road, Minhang District, Shanghai 200240, PR China; Yazhou Bay Institute of Deepsea Sci-Tech, Shanghai Jiao Tong University, No. 2 Road, Yazhou District, Sanya 572025, Hainan, PR China; State Key Laboratory of Microbial Metabolism, Joint International Research Laboratory of Metabolic & Development Sciences, School of Life Sciences & Biotechnology, Shanghai Jiao Tong University, 800 Dongchuan Road, Minhang District, Shanghai 200240, PR China; State Key Laboratory of Microbial Metabolism, Joint International Research Laboratory of Metabolic & Development Sciences, School of Life Sciences & Biotechnology, Shanghai Jiao Tong University, 800 Dongchuan Road, Minhang District, Shanghai 200240, PR China; State Key Laboratory of Microbial Metabolism, Joint International Research Laboratory of Metabolic & Development Sciences, School of Life Sciences & Biotechnology, Shanghai Jiao Tong University, 800 Dongchuan Road, Minhang District, Shanghai 200240, PR China; Archaeal Biology Center, Synthetic Biology Research Center, Shenzhen Key Laboratory of Marine Microbiome Engineering, Key Laboratory of Marine Microbiome Engineering of Guangdong Higher Education Institutes, Institute for Advanced Study, Shenzhen University, 3688 Nanhai Avenue, Nanshan District, Shenzhen 518055, PR China; State Key Laboratory of Microbial Metabolism, Joint International Research Laboratory of Metabolic & Development Sciences, School of Life Sciences & Biotechnology, Shanghai Jiao Tong University, 800 Dongchuan Road, Minhang District, Shanghai 200240, PR China; Yazhou Bay Institute of Deepsea Sci-Tech, Shanghai Jiao Tong University, No. 2 Road, Yazhou District, Sanya 572025, Hainan, PR China; State Key Laboratory of Microbial Metabolism, Joint International Research Laboratory of Metabolic & Development Sciences, School of Life Sciences & Biotechnology, Shanghai Jiao Tong University, 800 Dongchuan Road, Minhang District, Shanghai 200240, PR China; Yazhou Bay Institute of Deepsea Sci-Tech, Shanghai Jiao Tong University, No. 2 Road, Yazhou District, Sanya 572025, Hainan, PR China

**Keywords:** lytic proteins, prokaryotic viruses, endolysin, cell lysis, diversity and evolution, antimicrobial potential

## Abstract

Lytic proteins, essential for viral life cycles, mediate cell lysis, driving nutrient, and gene flow in ecosystems. Despite advances in understanding viral lysis mechanisms, the lytic proteins of prokaryotic viruses remain poorly understood at the macroevolutionary scale. Here, we constructed the Prokaryotic DNA Virus Lytic Protein Dataset, revealing the diversity, distribution patterns, and evolutionary drivers of lytic proteins across viral genomes. Our results demonstrate sequence and structural variation, suggesting that the composition of the lysis system is closely linked to viral genome size, host cell wall structure, and lifestyle, reflecting ecological adaptation. We observed that viral lytic proteins exhibit extensive sequence variation but retain structural conservation, suggesting a stronger selective pressure on structure that may be driven by the need to adapt and conform with specific cell envelope architectures. Phylogenetic analyses identified a significant co-evolutionary signal among lytic proteins, alongside extensive horizontal gene transfer of endolysin and holin encoding genes between bacteriophages and bacteria. These analyses also support that viral lytic proteins likely originated from bacterial sources, with different functional types having multiple independent origins. Moreover, comparative analysis of DNA and RNA virus lytic proteins demonstrates their diversity and differences across viral lineages. Revealing vast unexplored lytic proteins diversity, this study highlights their biotechnological potential against multidrug-resistant pathogens.

## Introduction

**Figure 1:**
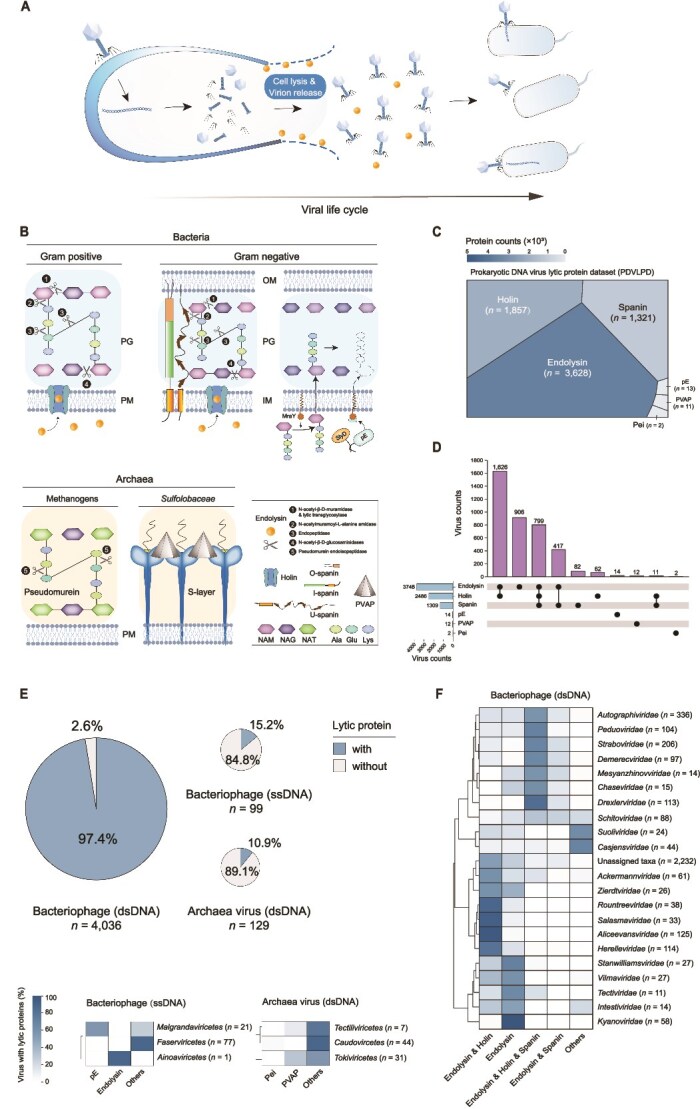
Overview of the PDVLPD. (A) Schematic representation of the lytic phase in the prokaryotic viral life cycle. Dots denote endolysins, which mediate viral particle release through cell wall degradation. For clarity, the diagram focuses exclusively on cell lysis and viral release, omitting other stages of the viral life cycle (e.g. adsorption, genome injection, and replication). (B) Mechanisms of LyP activity in diverse prokaryotic cell types. Key abbreviations: PG, peptidoglycan layer; OM, outer membrane; IM, inner membrane; PM, plasma membrane; pE, protein E. Major cell wall components, including N-acetylmuramic acid (NAM), N-acetylglucosamine (NAG), and N-acetyl-talosaminuronic acid (NAT), are labeled. Scissors and arrows indicate the target sites of distinct LyPs. The legend in the lower right corner categorizes LyPs and cell wall structural elements. Note: Component sizes are not to scale and are adjusted for visualization. (C) Composition of LyPs in the PDVLPD. Non-redundant LyP counts for each type are represented using a gradient color scale. (D) Distribution of LyP types. The bar chart depicts the number of viruses encoding specific LyP systems, whereas dots and connecting lines denote corresponding LyP types. The adjacent bar chart illustrates the number of viruses encoding each LyP type independently. (E) Distribution of LyPs across viral types. Pie chart areas correspond to the total number of viruses, with color segments representing the proportions of viruses encoding or lacking LyPs, respectively. (F) Distribution of LyPs across viral families. The heatmap indicates the percentage of viruses encoding LyPs within each family, with color intensity reflecting relative proportions. The total number of viruses per family is indicated in parentheses following the family name.

Current estimates indicate that ~10^31^ viral particles inhabit Earth, most being prokaryotic viruses infecting bacteria and archaea [1]. Due to their abundance, genetic diversity, and functional activity, viruses are pivotal components of global ecosystems [2]. Virus-induced lysis is the predominant fate of prokaryotic cells (Fig. 1A), second only to cell division [3], lysing ~20% of marine microorganisms daily [4], releasing 0.37–0.63 Gt of carbon annually [5]. This process was formerly regarded as a simple lysozyme-mediated event during phage’s maturation stage [3]. However, recent studies have demonstrated that this process is highly complex and tightly regulated, involving a suite of LyPs and diverse molecular mechanisms [3].

The mechanism of viral lysis in prokaryotic cells fundamentally involves disruption of the cell wall and membrane, shaped by structural differences between bacterial and archaeal cell envelopes (Fig. 1B). Bacterial cell walls primarily consist of peptidoglycan, with Gram-positive bacteria featuring thick multilayered peptidoglycan, and Gram-negative bacteria a thinner layer enveloped by an outer membrane [6]. Most archaeal cell walls comprise a surface layer (S-layer), whereas methanogenic archaea utilize pseudomurein-based structures [7, 8]. Double-stranded DNA (dsDNA) bacteriophages, among the most studied lysis models, employ a system comprising endolysin, holin, and spanin [9], these proteins work together in a coordinated manner to efficiently lyse bacterial cells [3]. Holin, the key regulator, integrates into the host membrane during the late phase of phage replication, forming pores that permit endolysin to access and degrade peptidoglycan [10, 11]. In Gram-negative bacteria, inner-membrane spanin (I-spanin) and outer-membrane spanin (O-spanin) form a complex bridging the periplasmic space, fusing inner and outer membranes to complete lysis [12]. In methanogenic archaea, pseudomurein endoisopeptidases (Pei) cleave peptide bonds within pseudomurein, compromising cell wall integrity [13]. For *Crenarchaeota*, virus-associated pyramid (VAP) structures transition from a closed to an open state, creating lytic pores in the host S-layer [14]. In contrast, single-stranded DNA (ssDNA) bacteriophage ϕX174 employs an indirect strategy. Its protein E (pE) inhibits MraY, a key enzyme in peptidoglycan synthesis, halting cell wall formation and inducing lysis [15]. Double-stranded RNA (dsRNA) viral endolysins (e.g. those from phiYY) exhibit bacterial cell wall degradation activity and antimicrobial potential [16]. Whereas single-stranded RNA (ssRNA) viruses rely on diverse single gene lysis (Sgl) proteins (e.g. Levivirus), further analyses have revealed diversity among Sgl proteins, necessitating systematic and comparative analysis [17]. These findings underscore the diverse and finely tuned mechanisms viruses employ to overcome host defenses, highlighting the evolutionary complexity of viral lysis pathways.

In recent years, virus-encoded endolysins have gained attention in medicine, food safety, environmental science, and agriculture for their rapid bactericidal activity and low resistance potential. PlySs2 effectively treats *Staphylococcus aureus* infections in clinical settings [18], whereas PlyP100 inhibits *Listeria monocytogenes* contamination in cheese for up to four weeks, maintaining activity under low-acid conditions [19]. In agriculture, endolysin from bacteriophages CMP1 and CN77 have shown potential in mitigating crop pathogens [20, 21]. Databases such as Phage Lytic Protein Database (PhaLP), integrating UniProt data with 16 095 entries support systematic discovery endolysins [22], and the VersaTile platform enables high-throughput engineering endolysin, generating ~10 000 variants and identifying a potent endolysin against *Acinetobacter baumannii*, validated in a porcine burn model [23]. Artificial intelligence (AI) has further accelerated endolysin discovery. DeepMineLys (CNNs-based) identified 16 endolysin candidates, with 11 confirmed enzymatically active [24], and DeepLysin discovered 17 novel endolysins, seven displaying potent *in vitro* activity and LLysSA9 demonstrating efficacy in mouse infection models [25]. These tools and platforms are transforming endolysin research, accelerating the discovery of novel antimicrobial agents.

Despite substantial advances in understanding virus-encoded endolysins over the past two decades, the diversity and functional mechanisms of prokaryotic viral lytic systems remain largely underexplored. In this study, we developed the prokaryotic DNA virus lytic protein dataset (PDVLPD), a rigorously curated resource guaranteeing high data quality through multi-step validation. Using this dataset, we systematically analyzed the sequence, structural diversity, and distribution patterns of LyPs, revealing the diverse strategies employed by prokaryotic viruses in selecting and combining these proteins. Our phylogenetic analysis identified horizontal gene transfer (HGT) events between viral LyPs and their prokaryotic homologs, underscoring the dynamic evolutionary interplay. Additionally, we discovered numerous uncharacterized LyPs within environmental microbiomes and assessed their potential for biotechnological applications. Furthermore, comparative analysis of DNA and RNA virus LyPs revealed their diversity and differences across viral lineages. This study provides valuable insights into the diversity and functional mechanisms of prokaryotic viral LyPs and establishes a crucial data foundation for future application and optimization through engineered modifications.

## Materials and methods

### Screening of prokaryotic DNA viral lytic proteins

To identify prokaryotic DNA viral LyPs, we developed four bioinformatics pipelines (Pipelines 1–4) with stringent quality control to construct the PDVLPD. Pipeline 1 retrieved LyP sequences from the following sources: endolysin (*n* = 4503) from the PhaLP (released on 25 June 2021) [22], holin (*n* = 748) from the Transporter Classification Database (TCDB, released in 2023) [26], spanin (*n* = 1109) from the Spanin Database (SpaninDB, released in 2018) [12], and pE (*n* = 5), Pei (*n* = 3), and protein forming Virus-Associated Pyramid (PVAP) (*n* = 15) sequences from the NCBI Reference Sequence Database (RefSeq, released in July 2023) [27]. Sequences were aligned with MAFFT (v7.455) [28] in automatic mode and used to construct Hidden Markov Models (HMMs) with HMMER (v3.4) [29]. The hmmsearch module (E-value ≤1e−10) [29] was employed to query all protein sequences from 4264 prokaryotic DNA viruses with complete genomes, sourced from the ICTV Virus Metadata Resource (VMR_MSL38_v2) database [30], the most authoritative resource for manually curated, high-quality viral genomes. Resulting hits were compiled into the prokaryotic viral LyP query dataset (Table S1). To ensure that viral LyPs were not missed due to divergent sequences or gene prediction limitations, we employed several additional steps to extract viral LyPs from the 4264 prokaryotic viral reference genomes. In Pipelines 2, LyP sequences were retrieved through keyword screening of annotated proteins from genomes classified by ICTV, using taxonomy-consistent entries from NCBI GenBank (released on July 2023) [27]. To account for limitations in predicting virus open-reading frames, we re-annotated the same set of genomes using Pharokka (v1.4.1) [31] with default parameters, including PHANOTATE [32] as its integrated ORF caller for gene prediction. Based on the same keyword list, we identified and filtered viral LyPs in Pipeline 3. To capture LyPs potentially missed by standard codon usage, we re-predicted open reading frames (ORFs) from the genomes of ICTV-classified prokaryotic viruses using Prodigal-gv (v2.11.0) [33] with default parameters, which evaluates all genetic codon tables without filtering. The resulting ORFs were subsequently scanned using the same HMM set from Pipeline 1 (E-value ≤1e−10), defining Pipeline four candidates. Before clustering, all candidate sequences from Pipelines 1–4 (including GenBank annotations, Pharokka re-annotations, and Prodigal-gv predictions) were pooled together and subject to additional curation.

### Quality control and PDVLPD construction

To ensure the accuracy and reliability of the LyP data, we referred to previously published quality control methods [12, 22, 25, 34]. Endolysin candidates were annotated with InterProScan (v5.66–98.0) [35] and excluded if identified as virion-associated lysins (VALs), which serve as structural components of viral particles, lacked enzymatically active domains (EADs), cell wall-binding domains (CBDs), or contained transmembrane regions (TMRs) (total filtered = 918). Endolysin proteins were functionally classified according to their domains, including glucosaminidase (cleaves β-1,4 glycosidic bonds between N-acetylglucosamine units), transglycosylase (cleaves β-1,4 glycosidic bonds between N-acetylmuramic acid and N-acetylglucosamine without hydrolysis), endopeptidase (targets peptide cross-links within stem peptides), muramidase (cleaves β-1,4 linkages between N-acetylmuramic acid and N-acetylglucosamine), amidase (cleaves the amide bond between muramic acid and stem peptides), s-CBD (single-domain cell wall binding domain), and m-EAD (multi-domain enzymatically active domain), and unclassified [3, 22, 25]. Holin candidates were analyzed with DeepTMHMM (v1.0.42) [36], excluding sequences without TMRs (removed = 1823), and classified by predicted TMRs count (TMRs-1 to TMRs-4) [34]. Spanin candidates were filtered by gene structure, removing sequences lacking TMRs for I-spanin, lacked a lipid box for O-spanin, or, for unimolecular spanin (U-spanin), either feature (removed = 887) [36]. Classification followed into SOS (separated outer membrane spanin), SIS (separated inner membrane spanin), EOS (embedded outer spanin), EIS (embedded inner spanin), OOS (overlapping outer spanin), OIS (overlapping inner spanin), and USP (unimolecular spanin) [12, 36]. The filtered LyP sequences underwent CD-HIT (v4.80) [37] at 100% identity to produce the final non-redundant PDVLPD, comprising six LyPs types: endolysin (*n* = 3626), holin (*n* = 1854), spanin (*n* = 1321), pE (*n* = 13), Pei (*n* = 2), and PVAP (*n* = 11) (Table S2).

### Viral lifestyle prediction

We identified 4264 complete prokaryotic viral genomes through the ICTV Master Species List (VMR_MSL38_v2; 2023 release) [30], retrieving assemblies and protein annotations from the NCBI GenBank [27] (accession list in Table S5). Lifestyle prediction was performed with PhaTYP (v2.0) [38] using default parameters, successfully classifying 3873 viruses.

### Host information of prokaryotic viruses

Host species information were obtained from NCBI Virus (released in July 2023) [39] via GenBank and RefSeq [27] identifiers, with data filtered for bacterial host criteria. Gram-staining classification was retrieved from BacDive (https://bacdive.dsmz.de/) [40]. Predicted hosts were identified by matching CRISPR spacers from RefSeq prokaryotic genomes (v2.12.0) to protospacers in viral genomes [41], following established methods [42]. CRISPR arrays were detected with Recognition Tool (CRT, v1.2) [43] using optimized parameters (*−*minRL 20, −maxRL 50, *−*minSL 20, *−*maxSL 60, *−*searchWL 7). According to prior studies [44], the ratio of spacer length to repeat length was constrained between 0.6 and 2.5, and CRISPR regions containing fewer than three spacers were excluded. Spacer sequences were aligned against phage genomes with BLASTn (v2.12.0) [45], retaining matches with ≥95% identity, ≥ 95% coverage, and ≤ 1 single nucleotide polymorphism (SNP). To evaluate potential taxonomic biases, random subsampling was performed at the phylum level: phyla with ≥100 distinct species were downsampled 100 species per phylum in each replicate (10 replicates total). For *Pseudomonadota*, *Actinomycetota*, and *Bacillota*, 2000 species were sampled per phylum in each replicate (also with 10 replicates). In each replicate, virus-host associations were re-inferred, and host range diversity quantified as the number of distinct bacterial species associated with each virus. Results represent means across all replicates, with comparative analyses among different lysis systems and functional endolysin types. Taxonomic annotation of bacterial and archaeal genomes was performed using the GTDB-tk (v2.3.2) [46].

### Protein sequence clustering

To analyze the diversity of LyPs, MMseqs2 (v16.7) easy-cluster was applied (50% identity, 90% coverage). Self-alignment were conducted using DIAMOND BLASTp (v2.0.14) [47] (E-value ≤1e-10), with amino acid sequence identity values as correlation indicators, each protein serving as both a source and a target node. Similarity networks were visualized in Cytoscape (v3.9.1) [48] with node colors denoting protein types. Representative sequences underwent average linkage clustering based on BLASTp LyPs identity values, and results were visualized as heatmaps using pheatmap (v1.0.12) [49] in RStudio (v4.2.2) [50].

### Protein structure prediction, comparison, and clustering

Protein structures of representative proteins were predicted using ColabFold (v1.5.2) [51], retaining only high-confidence structures (pLDDT ≥70). Structural comparisons were analyzed with Foldseek (v1.3) [52] easy-search to compute TM-score-based similarity matrices, followed by average linkage clustering and heatmap visualization in pheatmap (v1.0.12) [49] (RStudio v4.2.2) [50]. Using Foldseek (v1.3) [52] easy-cluster module with stringent parameters (alignment coverage ≥70%, TM-score ≥ 0.4, E-value <0.001), we clustered 779 representative endolysin structures, yielding 56 structural clusters and 40 singleton clusters. To establish structure–function relationships, we annotated conserved domains in the 56 clusters using InterProScan (v5.66–98.0) [35] (Pfam) and assigned to amidases, muramidases, peptidases, transglycosylases, glucosaminidases, s-CBDs, m-EADs, and unclassified groups. The relative proportions of each functional type within clusters were quantified and visualized as heatmaps using pheatmap201 (v1.0.12) in RStudio (v4.2.2) [50].

### Searching for distantly related lytic proteins based on protein structure

For viruses LyP lacking sequence based LyP similarity (131 dsDNA bacterial viruses, 84 ssDNA bacterial viruses, and 115 dsDNA archaeal viruses, totaling 16 747 protein sequences), MMseqs2 (v16.7) [53] was used with default parameters for clustering. Representative proteins were predicted with ColabFold (v1.5.2) [51], retaining only high confidence structures (pLDDT ≥70). A total of 3007 proteins with unknown functions were structurally aligned to PDVLPD representatives (874 endolysin, 525 holin, and 494 spanin proteins) using Foldseek (v1.3) [52] easy-cluster module (alignment coverage ≥70%, TM-score ≥ 0.4, E-value <0.001). Protein structures were visualized using PyMOL (v2.6) [54].

### Phylogenetic analysis of lytic proteins

Complete bacterial (*n* = 38 249) and archaeal (*n* = 2327) genomes were downloaded from GenBank (as of July 25, 2023) and analyzed using geNomad (v1.8) [55] with default parameters to remove 678 007 proviral regions, excluding viral fragments shorter than 4 kb were further filtered out to avoid potential interference with phylogenetic analysis caused by lytic genes in these short fragments. HMM models from PDVLPD were searched against non-proviral proteins using HMMER hmmsearch (v3.4) [29] (E-value ≤1e-10), identifying bacterial homologous endolysin (*n* = 66 693), holin (*n* = 14 524), and spanin (*n* = 71) from bacteria, as well as homologous endolysin (*n* = 256) and holin (*n* = 278) from archaea. Bacterial endolysin and holin homologs were clustered with MMseqs2 (v16.7) [53] (50% identity, 90% coverage); archaeal homologs and bacterial spanin were directly included in subsequent phylogenetic analyses. To reduce redundancy, TaxonKit (v0.18.0) [56] sampled sequences at the genus level, obtaining 639 and 505 representative sequences. The integrated LyP sequences were aligned using MAFFT (v7.0) [28], trimmed with trimAl (v2.0) [57]. A phylogenetic tree was constructed using IQ-TREE (v2.1.3) [58] with the MFP model, running 1000 bootstrap replicates for statistical validation. Finally, the phylogenetic tree was visualized using Chiplot [59].

To analyze the distribution of viral endolysin domains, we screened 66 693 bacterial and 256 archaeal homologous endolysins, retaining 55 022 and 167, respectively. For domain distribution analysis, we used the GTDB (release 220) [60] reference phylogenetic tree. We extracted EADs and CBDs from representative viral (*n* = 592 EAD, 149 CBD), bacterial (*n* = 548 EAD, 168 CBD), and archaeal (*n* = 178 EAD, 65 CBD) endolysins. These domains were aligned with MAFFT (v7.0) [28], trimmed with TrimAl (v2.0) [57], and used to construct separate maximum-likelihood phylogenetic trees in IQ-TREE2 (v2.1.3) [58] using the MFP model with 1000 bootstrap replicates. Trees were visualized using Chiplot [59].

### Cophylogenetic analysis of lytic proteins

To examine potential co-evolutionary relationships among endolysins, holins, and spanins, 38 conserved single-copy marker genes from 3872 *Caudoviricetes* viral genomes were identified using VOGDB HMMs [61, 62]. Multiple sequence alignment was performed using MAFFT (v7.0) [28], and trimmed with trimAl (v2.0) [57]. A phylogenetic tree of 2870 viruses was constructed using FastTree (v2.1.11) [63] with viral taxonomy classified according to ICTV (VMR_MSL40_v1) [64], and visualized in iTOL [65]. From this viral phylogenetic tree, we selected 561 viral genomes encoding all three LyPs (endolysin, holin, and spanin) to construct a sub-phylogenetic tree and separate trees for each of the three protein classes. Phylogenetic congruence was assessed via PACo (v0.4.2) [66] in R package and ParaFit (v5.8.1) [67] in ape package [68], each with 999 permutations and incorporated Cailliez correction, visualized using phytools (v2.4.4) [69].

### HGT analysis

We referred to previously published methods for HGT analysis of prokaryotic viral proteins [70]. PDVLPD proteins were self-aligned with DIAMOND BLASTp (v2.0.14) [47], LyPs retaining pairs with identity ≥95%. Homologous bacterial (≥ 90% identity) were identified, whereas no archaeal proteins were detected. Viral and bacterial LyPs were then clustered using MCL (v22–282) [71] (Inflation = 2.0), requiring at least two proteins per cluster from both sources. Next, multiple sequence alignments for each cluster were performed using MAFFT (v7.0) [28], trimmed using trimAl (v2.0) [57], and maximum likelihood phylogenetic trees for each cluster were then constructed using PhyML (v3.0) [72] (LG model). Tree reliability was assessed using CONSEL (v0.2) [73] with an approximately unbiased (*P* ≥ 0.05) test. Phylogenetic trees that passed this test were retained for subsequent donor and recipient analysis. Specifically, each tree was rooted using the midpoint method, according to prior studies [70], the donor was identified as the protein at the basal position of the branch, whereas the recipient was the protein at the terminal position. HGT networks were constructed by mapping tree proteins as nodes, with edge lengths defined by amino acid identity, visualized in Cytoscape (v3.9.1) [48].

### Diversity assessment of unexplored lytic proteins

Using PDVLPD HMM profiles, HMMER hmmsearch (v3.4) [29] (E-value ≤1e-10) was applied to search IMG/VR v4 – high-confidence genomes only (released on September 20, 2022) [74] for viral protein sequences. Abundant endolysin, holin, and spanin sequences were clustered with MMseqs2 (v16.7) [53] (50% identity, 90% coverage), and representatives selected for downstream analysis; all pE, Pei, and PVAP sequences were included in the analysis. A sequence similarity network was constructed from HMMER hmmsearch (v3.4) [29] scores with each protein defined as both source and target node, visualized in Cytoscape (v3.9.1) [48].

### Saturation evaluation of lytic proteins

To assess LyPs diversity, saturation curves were constructed from three perspectives: sequence, functional, and structural diversity. Sequence diversity was evaluated from representative clusters generated by MMseqs2 (50% identity, 90% coverage). Functional diversity was analyzed by self-alignment of representative proteins followed by MCL (v22–282) [75] (inflation = 2.0) clustering to define functional groups. Structural diversity was evaluated using Foldseek easy-cluster (coverage ≥70%, TM-score ≥ 0.4, E-value <0.001) [52]. Saturation curve data were obtained using random sampling statistics implemented in a Python script (sample range: 0 to 45 000; step size: 500; repetition frequency: 10 times per group), visualized in ggplot2 (v3.4.3) [76].

### Analysis of lytic proteins targeting WHO priority pathogens

To evaluate the application potential of viral lytic enzymes against drug-resistant pathogens, we analyzed endolysin hmmsearch data from IMG/VR v4 viral genomes (see “Diversity Assessment of Unexplored LyPs”) and from complete and chromosomal-level bacterial genomes in GenBank (downloaded on July 25, 2023; see “Phylogenetic Analysis of LyPs”). By integrating host information, we identified phage endolysin hmmsearch data corresponding to eight WHO priority bacterial genera: *Escherichia, Salmonella, Klebsiella, Staphylococcus, Mycobacterium, Pseudomonas, Acinetobacter,* and *Enterococcus.* A custom script filtered, classified, and extracted phage endolysin sequences specific to these bacterial genera. Experimentally validated endolysin data were obtained from the UniProt [77] and Swiss-Prot [78]. Using hmmsearch (v3.4.1) scores from Swiss-Prot, IMG/VR v4, GenBank, and PDVLPD as correlation indicators, proteins from different data sources were defined as source and target nodes to construct a sequence similarity network, visualized in Cytoscape (v3.9.1) [48].

### Identification and diversity analysis of prokaryotic RNA viral lysis proteins

Utilizing the same data source (ICTV VMR_MSL38_v2; 2023 release) [30] and retrieval strategy as for the PDVLPD, we incorporated dsRNA viruses (*Vidaverviricetes*) and ssRNA viruses (*Leviviricetes*) prokaryotic viruses. A total of 889 complete RNA viral genomes (seven dsRNA and 882 ssRNA) were screened, and protein sequences were obtained from NCBI GenBank (Release April 2025) [27]. Representative protein structures were predicted using ColabFold (v1.5.2) [51], LyPs yielding 3158 ssRNA viral LyPs, of which 2088 had high-confidence structural models (pLDDT ≥70). Using 35 experimentally validated lysis peptides as positive controls [17], we selected 30 Sgl peptides with reliable structural predictions as positive references. Structural clustering analysis was performed using the Foldseek easy-cluster module (coverage ≥70%, TM-score ≥ 0.4, and E-value <0.001) [52], and clustered putative Sgl structures were subsequently analyzed using TM-align [79] with structural similarity evaluated based on TM-score, chosen for its suitability in comparing protein structures with low sequence similarity, and its avoidance of k-mer generation errors that may occur with Foldseek [52] easy-search when processing short sequences. To compare sequence diversity between DNA and RNA viral LyPs, we performed DIAMOND BLASTp (v2.0.14) [47] self-alignment of the integrated dataset (PDVLPDLyPs and RNA viral LyPs identified in this study) with an E-value ≤1 × 10^−5^. Amino acid sequence identity values were used to construct a sequence similarity network, with each protein represented as a node and visualized using Cytoscape (v3.9.1) [48].

## Results

### Construction of the PDVLPD

Utilizing prokaryotic viral genome sequences from the International Committee on Taxonomy of Viruses (ICTV, VMR_MSL38_v2) database [30], we developed four bioinformatics pipelines (Pipelines 1–4) with stringent quality controls (detailed in Methods) to construct the PDVLPD (Fig. S1). This dataset includes endolysin (*n* = 3626), holin (*n* = 1854), spanin (*n* = 1321), pE (*n* = 13), Pei (*n* = 2), and PVAP (*n* = 11) (Fig. 1C and Table S2). Sourced exclusively from complete viral genomes obtained from authoritative databases, the dataset incorporates rigorous candidate screening protocols to ensure reliability and accuracy. PDVLPD integrates all currently recognized LyP types, providing a comprehensive view of prokaryotic DNA virus lysis system.

Distribution analysis showed that the endolysin & holin combination is the most prevalent lytic mechanism (38.1%, *n* = 1626), followed by endolysin alone (21.2%, *n* = 906) and the three-component endolysin and holin and spanin system (18.7%, *n* = 799); standalone holin and spanin are rare (1.5%, *n* = 82; 2.0%, *n* = 62) (Fig. 1D). Taxonomically, 97.4% of dsDNA bacteriophages encode at least one LyP, versus 15.2% of ssDNA bacteriophages—driven by dominant *Inovirus* (*n* = 79) that release progeny without cell lysis [80]. Only 10.9% of 129 archaeal dsDNA viruses encode detectable LyPs (Fig. 1E), likely reflecting limited characterization of archaeal viral lytic mechanisms and proteins [81].

Viral groups exhibit specific preferences for LyPs and combinations, reflecting diverse strategies for host cell lysis. Certain dsDNA viral families demonstrate conservation in their lysis systems (Fig. 1F). Single-component endolysin predominates in *Stanwilliamsviridae*, *Tectiviridae*, and *Kyanoviridae*. The two-component endolysin & holin system is prevalent in *Rountreeviridae*, *Salasmaviridae*, and *Aliceevansviridae*. In addition, the three-component endolysin & holin & spanin system is characteristic of *Peduoviridae*, *Straboviridae*, *Demerecviridae*, and *Drexlerviridae*. pE is exclusive to *Alphatrevirus* within *Malgrandaviricetes*. Additionally, endolysin was identified in the ssDNA bacteriophage *Finnlakevirus* FLiP (*Ainoaviricetes*), suggesting that ssDNA viruses may also use endolysin for host cell lysis, consistent with recent studies [82]. Among dsDNA archaeal viruses, Pei is primarily associated with *Caudoviricetes* infecting methanogenic archaea [83]. We observed that PVAP, which was previously reported only in *Tokiviricetes* [81], was identified in *Tectiliviricetes* (non-tailed viruses), expanding its known distribution. These patterns underscore diverse lytic strategies and highlight non-tailed bacteriophages and archaeal viruses are also reservoirs of lytic mechanisms.

### Sequence diversity and structural conservation of lytic proteins

**Figure 2:**
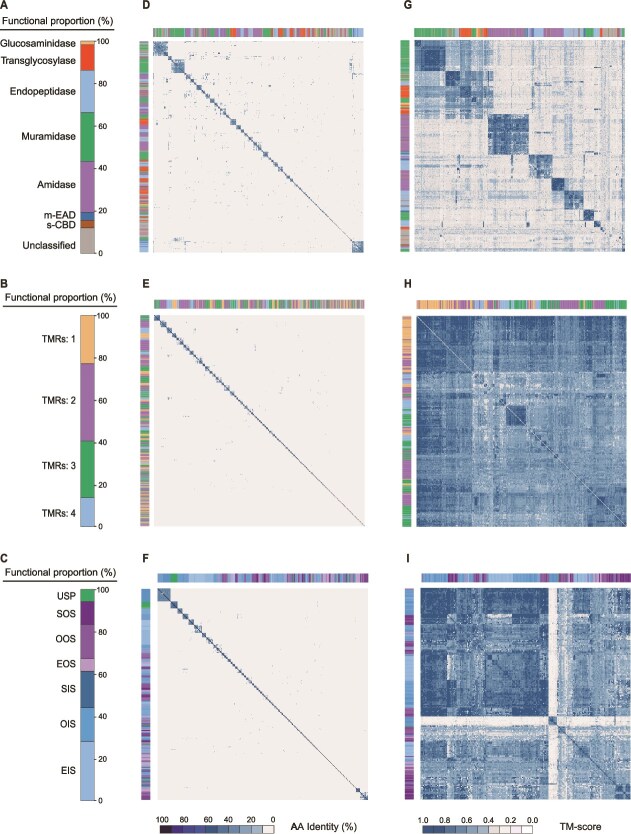
Sequence and structural similarity analysis of prokaryotic viral lytic proteins. (A–C) Proportions of different functional or structural categories within the three major LyP types. Endolysins (A) are classified by enzymatic function (glucosaminidase, transglycosylase, endopeptidase, muramidase, amidase, s-CBD, m-EAD), holins (B) by the number of transmembrane regions (TMRs: 1, 2, 3, 4), and spanins (C) by gene structure categories (SOS, SIS, EIS, EOS, OIS, OOS, USP). (D–F) Sequence similarity matrices of prokaryotic viral LyPs. From top to bottom, matrices and category distributions are shown for endolysins (*n* = 883) (D) holins (*n* = 525) (E) and spanins (*n* = 427). (F) Matrices were constructed using pairwise amino acid sequence comparisons, with color intensity reflecting sequence similarity. Side color bars indicate the distribution of LyP categories. (G–I) Structural similarity matrices of prokaryotic viral LyPs. Matrices were generated using Foldseek [52] to compare predicted protein structures for endolysins (*n* = 779) (G), holins (*n* = 326) (H), and spanins (*n* = 238) (I) based on template Modeling score (TM-score) alignment. TM-score is a normalized metric (range: 0–1) for assessing structural similarity, commonly used in protein comparison due to its robustness across varying sequence lengths [79]. Color intensity represents structural similarity, with side color bars indicating category distributions. Only LyPs with reliable predicted structures (pLDDT ≥70) were included in this analysis.

To elucidate the diversity of prokaryotic viral LyPs, we first examined functional domain composition within each protein type (Fig. 2A–C). For endolysin, amidase (24.0%) and muramidase (23.0%) were most frequent, followed by endopeptidase (20.0%), transglycosylase (12.0%), m-EAD (3.7%), s-CBD (3.6%), and glucosaminidase (1.7%) (Fig. 2A). Holin diversity was mainly shaped by the number of transmembrane regions (TMRs), with TMR2 being the most common (36.7%), followed by TMR3 (26.8%), TMR1 (22.8%), and TMR4 (13.7%) (Fig. 2B). Spanin functional types showed a broader spread: OIS (25.9%), SIS (15.9%), EIS (14.7%), SOS (10.0%), OOS (14.9%), EOS (5.6%), and USP (5.3%) (Fig. 2C). Then, we constructed a sequence similarity network (Fig. S2) and clustering matrix (Fig. 2, D to F) using representative PDVLPD sequences, which revealed <20% average sequence similarity between functional categories, indicating high sequence divergence. Endolysins exhibited the highest normalized connectivity (average degree = 25.93), exceeding that of holins (9.82) and spanins (9.08), suggesting greater modularity and functional redundancy in endolysin evolution. In the endolysin network (*n* = 883), sequences clustered by functional domains—including glucosaminidase, transglycosylase, endopeptidase, muramidase, amidase, s-CBD, and m-EAD—whereas maintaining certain interrelationships (Fig. S2). A Pei protein [81] (Pei_1) from *Methanobacterium* virus C158 (*Caudoviricetes*) shared 43.9% identity with EL_1687 from *Actinomycetota*-infecting bacteriophages (Table S2), with template modeling score (TM-score) of 0.776 overall and 0.956 for the EAD (Fig. S3, A to C). In contrast, PeiP from *Methanobacterium* phage psiM2 formed a singleton. Meanwhile, Holin (*n* = 520) and spanin (*n* = 426) (Fig. S2) networks exhibited fewer interconnections, reflecting higher sequence diversity. Conversely, pE (*n* = 14) and PVAP (*n* = 12) displayed greater conservation: *Alphatrevirus* pEs clustered within *Malgrandaviricetes*, whereas 11 *Tokiviricetes* PVAPs formed a distinct cluster (barring one *Tectiliviricetes* outlier). These results highlight both the sequence and structural diversity of LyPs across prokaryotic viruses, with conserved features suggesting functional or evolutionary constraints.

Representative proteins from each sequence cluster underwent structure prediction and structural clustering analysis using the TM-score metric (Fig. 2G–I). Structural clustering of endolysin revealed distinct groupings based on enzymatic function (Fig. 2G). Muramidases, transglycosylases, and glucosaminidases, which target β-1,4-glycosidic bonds, formed a single cluster. Amidases, which hydrolyze amide bonds, and endopeptidases, which cleave peptide bonds, were distributed across two and three clusters, respectively. m-EAD co-clustered with amidases and endopeptidases, whereas s-CBD exhibited greater structural diversity without a clear clustering pattern. For holin, structural similarity was driven by TMR number (Fig. 2H), highlighting their conserved membrane-associated architecture. Spanin clustering reflected conservation based on cellular membrane localization (Fig. 2I), forming two major groups comprising I-spanin and O-spanin, with an additional smaller cluster containing OIS. Structural clustering covered fewer units than sequence clustering: endolysins (96 vs 779 clusters; avg 8.11, max 66), holins (95 vs 326), and spanins (63 vs 238). Some endolysin structural clusters spanned multiple enzymatic types (Fig. S4). Endolysin structural clusters demonstrate functional specificity alongside multifunctionality, with conserved domains exhibiting substructural differentiation. Their structures correlate strongly with enzymatic mechanisms yet retain cross-functional similarities, indicating both convergent and divergent evolutionary pathways (Fig. S5). These findings underscore that despite extensive sequence diversity, LyPs exhibit significant structural conservation, with clustering patterns closely aligned with their enzymatic functions and membrane localization.

The pronounced structural conservation among LyPs highlights structural similarity for identifying unrecognized viral LyPs missed by sequence-based annotation methods. Structural clustering revealed that conserved functional folds and catalytic activities (e.g. muramidase, transglycosylase, endopeptidase, amidase) can be identified even at 0% sequence identity (Fig. S6). Representative cases include proteins annotated only at the superfamily level or lacking catalytic annotation, yet showing high structural similarity (TM-score = 0.498–0.745) to cluster representatives with defined functions, supporting confident functional assignments (muramidase GH_24, SLT_related transglycosylase, PET_M15 endopeptidase, Ami_2 amidase). These results confirm that 3D structural analysis can transcend sequence limitations to provide crucial functional predictions for uncharacterized proteins. We subsequently performed alignment using representative LyPs from the PDVLPD dataset against 6656 proteins annotated as “unknown” or “hypothetical” from viruses lacking previously identified LyPs. The dataset comprised 131 dsDNA bacteriophages, 84 ssDNA bacteriophages, and 115 dsDNA archaeal viruses. This analysis identified 36 putative endolysins, 152 putative holins, and 15 putative spanins across 95 dsDNA viruses and two ssDNA viruses, including 49 bacteriophages and 48 archaeal viruses (Table S3). Newly identified LyPs span all major functional categories, expanding the known diversity of LyPs, particularly in archaeal viruses. Representative examples include a PET_C39-like endopeptidase from *Methanophagales* virus GBV302 (TM-score = 0.556), holins from *Lokiarchaeia* virus SkuldV1 (TMRs: 2, TM-score = 0.460) and *Halorubrum* pleomorphic virus 9 (TMRs: 3, TM-score = 0.410) (Fig. S3 and Table S3). These results indicate that archaeal viruses may encode LyPs with substantial structural similarity to bacteriophage counterparts despite low sequence homology. Experimental validation will be necessary to confirm the enzymatic activity of these putative archaeal LyPs.

### Ecological drivers of viral lytic protein diversity

**Figure 3:**
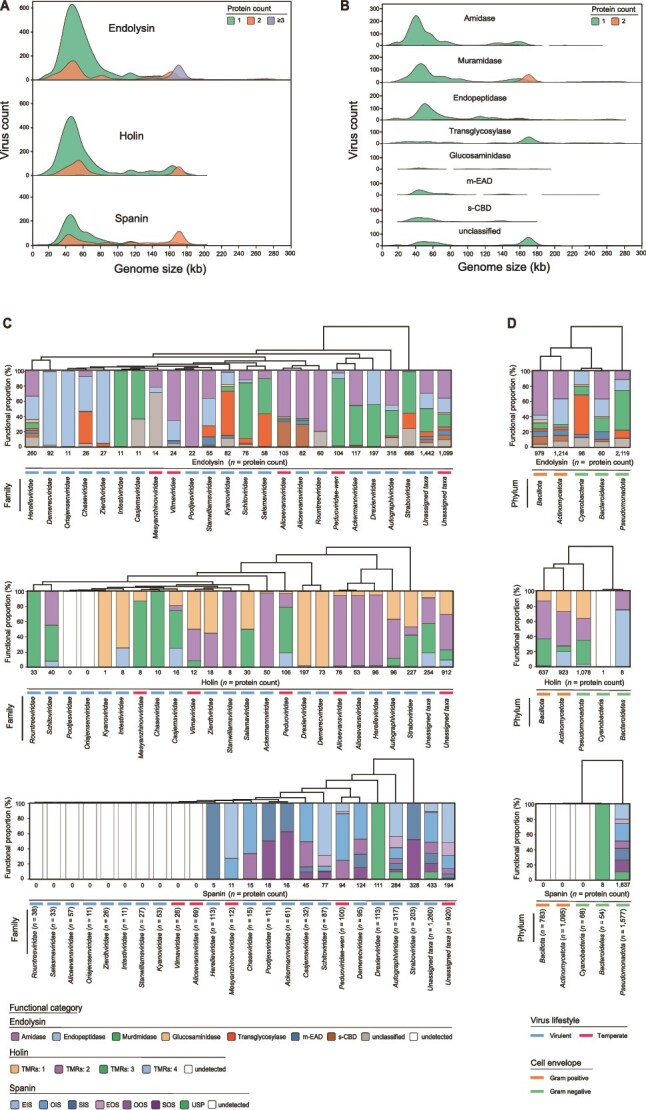
Distribution patterns of lytic proteins in prokaryotic viruses. (A) Association of LyP distribution with viral genome size. Colors represent the frequency of each LyP type occurring in individual viral genomes at varying copy numbers. (B) Distribution of endolysin functional types relative to viral genome size. Colors indicate the frequency of endolysins occurring in individual viral genomes at different copy numbers. (C) Composition of LyPs in viruses with distinct lifestyles. The stacked bar chart depicts the proportion of functional or structural categories of LyPs within each viral family. The total number of LyPs per family is annotated below the *X*-axis. Viral lifestyles are indicated by the bottom color bar (right bar, temperate viruses; left bar, virulent viruses). Clustering was performed using the complete linkage method. (D) Composition of LyPs in viruses infecting different bacterial phyla. The stacked bar chart shows the proportion of functional or structural categories of LyPs in viruses infecting each bacterial phylum. The total number of LyPs per bacterial phylum is labeled below the *X*-axis. Color bars represent viruses infecting gram-positive and gram-negative bacteria, respectively. Clustering was performed using the complete linkage method.

To investigate factors influencing LyP distribution in prokaryotic viruses, we analyzed the relationship between viral genome size and LyP-encoding gene prevalence (Table S4). A significant positive correlation was observed between genome size and LyP copy number (Fig. 3A), with the strongest relationship for endolysin (*r* = 0.51, *P* < 0.001), followed by spanin (*r* = 0.43, *P* < 0.001), and a weak but statistically positive correlation with holin (*r* = 0.13, *P* < 0.001) (Fig. 3A). In viruses with 20–80 kb genomes, single endolysin genes predominate, primarily of amidase, muramidase, and endopeptidase types (74% of types). Besides, m-EAD and s-CBD also feature prominently. In contrast, viruses with 80–140 kb genomes predominantly encode endopeptidases, with other types rare. In the 140–200 kb range, functional diversity increases, with amidase, muramidase, transglycosylase, and unclassified enzymes forming major groups (Fig. 3B). Holin and spanin types vary with genome size, showing distinct distribution patterns across size ranges (Fig. S7). Correlation analysis further revealed that genome size is strongly and positively correlated (*r* > 0.37, *P* < 0.001) with certain LyP types, including muramidase- and amidase-type endolysins, 1-TMR and 2-TMR holins, and SOS, OOS, and OIS-type spanins, whereas other types showed weak or no correlation (Fig. S8). These findings highlight that viral genome size strongly influences LyP frequency and functional diversity, reflecting adaptive strategies employed by viruses with varying genome sizes in selecting lytic mechanisms.

We predicted viral lifestyle (virulent or temperate) and examined their correlation with lytic strategies (Fig. 3C). LyPs showed an interspersed distribution between lifestyles, suggesting that viral lifestyle is not the primary determinant of lysis strategy. However, some categories show distinct lifestyle preferences. Transglycosylase endolysins are absent in most temperate viruses but are prevalent among virulent viruses, particularly in *Kyanoviridae*, *Salamaviridae*, and *Chaseviridae*. Additionally, USP-type spanin was absent in temperate viruses but widely distributed in virulent viruses, with *Drexlerviridae* exhibiting 100% prevalence (Fig. 3C). These findings suggest that although viral lifestyle does not strictly dictate lytic strategies, specific LyP types show marked associations with virulent or temperate lineages.

The distribution patterns of LyPs in bacteriophages targeting Gram-positive and Gram-negative bacteria highlight the influence of host cell wall architecture on viral lysis strategies (Fig. 3D). Among bacteriophages infecting Gram-positive hosts, amidases and endopeptidases dominate—particularly amidase in *Bacillota/Actinomycetota* and endopeptidase in *Actinomycetota*. In contrast, Gram-negative phages show transglycosylase dominance in *Cyanobacteria* versus muramidase prevalence in *Pseudomonadota*, diverging sharply from Gram-positive patterns. Holins are equally distributed in bacteriophages of both Gram-positive and Gram-negative hosts. Conversely, Spanin distribution reflects cell wall differences: absent in Gram-positive phages (no outer membrane) and *Cyanobacteria* phages, whereas *Pseudomonadota* phages encode all spanin types and *Bacteroidetes* phages exclusively use USP. These findings underscore phage lysis adaptability to host structural constraints.

Lysis is a pivotal stage in the viral life cycle, potentially influencing host range. To investigate this, we conducted a comparative analysis of host range variations associated with different lytic systems and endolysin functional classes. Viral hosts were predicted based on CRISPR spacer-protospacer sequence similarity (as described in the Methods section). Viruses employing endolysin and holin or endolysin and holin and spanin systems exhibited significantly broader host ranges (median = 2, *P* < 0.01) than those with endolysin alone or endolysin and spanin systems (Fig. S9A). Similarly, viruses encoding muramidase-, amidase-, or glucosaminidase-type endolysins (median = 2) had broader host ranges than those encoding endopeptidase, transglycosylase, m-EAD, or s-CBD types (median = 1) (Fig. S9B). Subsampling analyses to account for uneven taxonomic representation yielded variable results; however, patterns remained largely consistent when restricted to the three most abundant phyla—*Pseudomonadota*, *Actinomycetota*, and *Bacillota* (Fig. S9C–D), suggesting that although absolute correlation values may be influenced by database taxonomic bias, the overall trends remain robust within dominant bacterial lineages. These host range differences likely reflect the target specificities of different endolysins: muramidase-, amidase-, and glucosaminidase-type endolysins typically target highly conserved bonds in bacterial peptidoglycan backbones (e.g. MurNAc–GlcNAc, MurNAc–L-Ala) [84, 85], which exhibit broad conservation across most bacterial phyla; in contrast, endopeptidase-type endolysins target peptide bonds that vary substantially among different bacterial taxa [86]. The differential prevalence of these target structures among bacteria may represent a key determinant underlying the observed variations in host range for viruses encoding distinct endolysin types.

### Evolutionary origins and disseminations of lytic proteins

**Figure 4:**
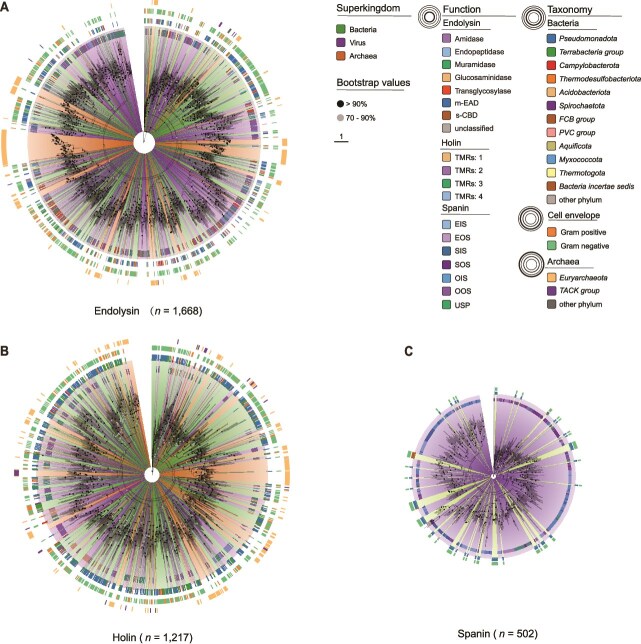
Phylogenetic analysis of prokaryotic viral lytic proteins. (A) Phylogenetic tree of endolysins, comprising 773 viral sequences, 639 bacterial sequences (representative sequences sampled from 639 genera), and 256 archaeal sequences. (B) Phylogenetic tree of holins, comprising 497 viral sequences, 505 bacterial sequences (representative sequences sampled from 505 genera), and 215 archaeal sequences. (C) Phylogenetic tree of spanins, comprising 431 viral sequences and 71 bacterial sequences. All trees were constructed using the MFP model in IQ-TREE (v2.13) [58], with 1000 bootstrap replicates for branch support evaluation. Scale bar indicates one amino acid substitution per site. Tree visualization and annotation were performed using Chiplot [59]. To manage the extensive number of bacterial homologous LyPs and ensure a structured phylogenetic representation, bacterial sequences were categorized by taxonomic levels using TaxonKit (v0.18.0) [56]. Branches are color-coded to indicate the source domain of LyPs (viruses, bacteria, archaea). Outer rings display taxonomic classification (phylum level for archaea and bacteria), bacterial gram classification, and LyP categories. Node colors represent bootstrap support values, with nodes exceeding 90% support highlighted in black.

To elucidate evolutionary relationships between virus-encoded LyPs and their prokaryotic homologs, we performed a homology search across 38 249 bacterial and 2327 archaeal genomes using profile HMMs constructed from 3626 non-redundant LyPs derived from the PDVLPD dataset. This analysis identified plenty of bacterial homologs of endolysin (*n* = 66 693), holin (*n* = 14 524), and spanin (*n* = 71); archaeal homologs of endolysin (*n* = 256) and holin (*n* = 278). Phylogenetic analyses of representative sequences revealed extensive intermingling of viral, bacterial, and archaeal lineages for endolysin and holin, consistent with frequent HGT (Fig. 4). Bacterial homologs were predominantly found in *Pseudomonadota* and *Terrabacteria*, whereas archaeal homologs in *Euryarchaeota* and *TACK* groups. Viral sequences were interspersed within these clades, suggesting prokaryotes as key contributors to viral LyPs diversification. In contrast, the spanin phylogeny lacked archaeal homologs, indicating a distinct evolutionary path. Functional analysis of endolysin homologs highlighted taxon-specific HGT patterns (Fig. S10). These findings underscore the extensive horizontal transfer of LyP-encoding genes, particularly between viruses and bacteria, revealing a dynamic interplay that has shaped the evolution of lysis machinery.

To investigate the origins of LyPs, we analyzed endolysin domain-level distribution and phylogeny. Bacterial endolysin domains exhibit greater diversity and abundance than archaeal counterparts (Fig. S11A), suggesting their higher likelihood as evolutionary sources. Specifically, amidase domains (Ami_2, Ami_3), transglycosylase domain (SLT_related), and endopeptidase domains (PET_M23, NLPC_P60) were widely distributed in bacteria, whereas other domains (Ami_5, LT_GEWL_like, PET_M15, PET_C70, PET_C39, CHAP) showed sparse distribution with certain bacterial lineage preferences. In contrast, all muramidase and glucosaminidase domains were sparse. Phylogenetic analysis of EADs and CBDs (Fig. S11B–C) revealed similarly intertwined evolutionary patterns between these domains and full-length enzymes. These findings suggest complex origins of viral endolysins, with potential divergent evolutionary pathways: some (e.g. transglycosylases, amidases, endopeptidases) likely originated from bacteria, whereas others (e.g. muramidases, glucosaminidases) may have evolved within viruses. HGT may explain virus-specific or dominant domains retained in viral genomes but lost in hosts, and archaeal contributions may be underestimated due to limited genomic data.

**Figure 5:**
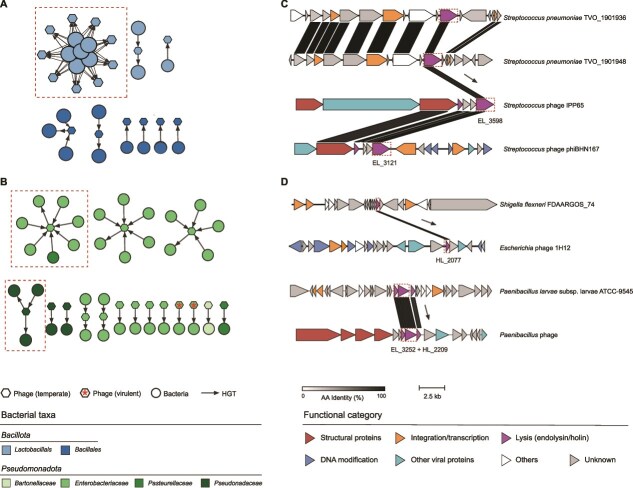
HGT of lytic proteins between bacteriophages and bacteria. (A and B) HGT events involving LyPs between bacteriophages and bacteria from the phyla *Bacillota* (A) and *Pseudomonadota* (B). Hexagonal and circular nodes represent bacteriophages and bacteria, respectively. Node colors indicate taxonomic classification (phylum and family levels). Arrows between nodes denote the inferred direction of gene transfer based on phylogenetic analysis, with asterisks marking lytic phages. Dashed boxes highlight HGT events further detailed in panels. (C) HGT events involving endolysin genes. (D) HGT events involving both holin and endolysin genes. Genes are depicted as arrows, with color and direction indicating gene function and transcriptional orientation, respectively. Connections and shaded areas between genes represent amino acid sequence similarity, visualized using Clinker [101]. Functional categories are color-coded, and protein identifiers from the PDVLPD are annotated adjacent to corresponding genes.

After characterizing LyP domain distribution and phylogeny, we conducted cophylogenetic analyses of 561 *Caudoviricetes* genomes encoding all three LyP classes (endolysins, holins, and spanins). Species and protein family trees were reconstructed and compared using PACo [66] and ParaFit [67] analyses, revealing significant topological concordance between each LyP family and their host viruses (PACo: *P* = 0; ParaFit: *P* = 0.001; Fig. S12), as well as among the three LyP families themselves (PACo: *P* = 0; ParaFit: *P* = 0.001). These results demonstrate long-term co-evolution among *Caudoviricetes* LyPs, suggesting this evolutionary interplay contributes to the formation of highly efficient, complex lysis systems. To validate the hypothesis of HGT in LyP evolution, we constructed an HGT network to trace gene exchange between bacteriophages and bacteria (Fig. 5A and B). The analysis identified recent HGT events primarily between *Pseudomonadota* and *Bacillota*, likely reflecting a bias of the abundance of genomic data for these groups in the public database. Most observed HGT events were unidirectional, with bacterial genes transferring to temperate bacteriophages. Examples include endolysin transfer from *Streptococcus pneumoniae* TVO_1901948 to *Streptococcus* phage IPP65 (92% identity) and holin transfer from *Shigella flexneri* FDAARGOS_74 to *Escherichia* phage 1H12 (90.5% identity), with the latter subsequently found in *Escherichia coli* DORAA 514_21 (90.5% identity), indicating reverse transfer from phage to bacterium. Multi-gene transfers were also observed, e.g. *Paenibacillus* phages acquired both endolysin and holin genes from *Paenibacillus larvae* subsp. larvae ATCC-9545 (Fig. 5D). The inferred directionality of HGT events in this study should be regarded as tentative due to limitations in rooting the phylogenetic trees (high sequence similarity and absence of appropriate outgroups). Nevertheless, these findings reveal characteristic bidirectional flow of lysis genes between phages and bacteria, highlighting the complex interaction mechanisms underlying the evolution of lysis systems.

### Unexplored diversity and application potential of lytic proteins

**Figure 6:**
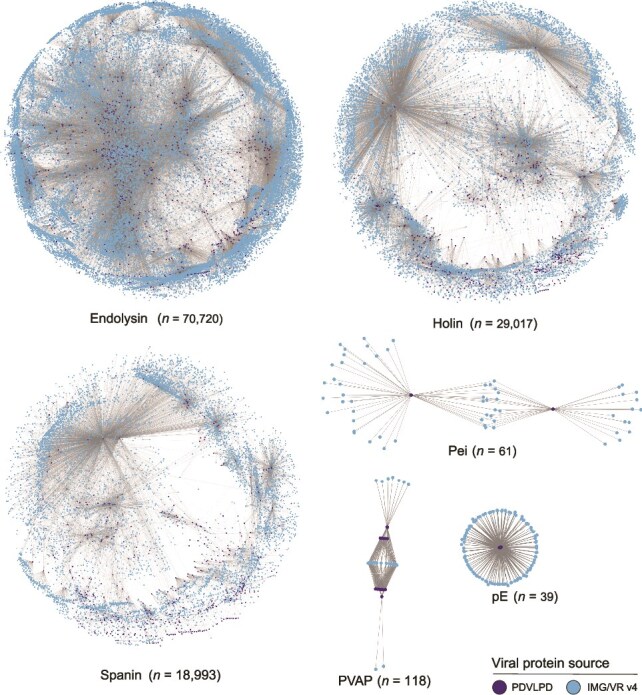
Global diversity of lytic proteins encoded by uncultured viruses. Due to the extensive number of endolysin, holin, and spanin sequences, clustering was initially performed, followed by the selection of representative sequences for sequence similarity network construction. In contrast, the networks for Pei, PVAP, and pE were constructed using all available sequences due to their limited abundance. Nodes represent LyP sequences, with color-coded nodes denoting sequences derived from PDVLPD and IMG/VR v4, respectively. Edges connecting nodes represent sequence similarity, with edge length inversely proportional to the degree of similarity.

Given that PDVLPD was constructed from a rigorously curated viral database, its reliability is high but coverage is limited-especially for uncultivated environmental viruses. We further analyzed the Integrated Microbial Genomes and Viruses Resource (IMG/VR v4) [74] database, which contains over 5 202 181 viral sequences. This search identified more than 1.3 million LyP, including endolysin (*n* = 724 636), holin (*n* = 344 718), spanin (*n* = 261 425), pE (*n* = 21), Pei (*n* = 203), and PVAP (*n* = 102), expanding known counts by more than 100-fold (Fig. 6). Saturation curve analysis revealed distinct diversity patterns across lytic components (Fig. S13). MMseqs2 clustering yielded 70 720 endolysin, 29 017 holin, and 18 993 spanin representatives. Holin and spanin sequence diversity approached saturation, whereas endolysins remained undersampled (Fig. S13A). Subsequent MCL clustering revealed convergence: endolysins formed only 164 clusters despite their high sequence count, compared to 260 holin and 289 spanin clusters (Fig. S13B), indicating most endolysins belong to conserved functional families, whereas holins/spanins exhibit greater sequence-level diversification. Structural clustering reinforced this trend: holins showed the highest structural variability (95 clusters from 326 representatives), exceeding endolysins (96 clusters/779 reps) and spanins (63 clusters/238 reps) (Fig. S13C). All three approached structural saturation, confirming holins as the most functionally diverse lytic component. In contrast, Pei, pE, and PVAP showed modest increases in identified sequences compared to the PDVLPD database, reflecting their limited distribution among known viral populations (Fig. 6). These findings underscore the extensive but uneven distribution of LyP diversity across viral genomes, highlighting the need for further exploration of underrepresented LyPs and their functional roles in viral lysis mechanisms.

Endolysins offer a promising approach to combat drug-resistant pathogens. To assess their therapeutic potential, we analyzed bacteriophage genomes targeting eight priority drug-resistant bacterial genera identified by the World Health Organization (WHO): *Escherichia*, *Salmonella*, *Klebsiella*, *Staphylococcus*, *Mycobacterium*, *Pseudomonas*, *Acinetobacter*, and *Enterococcus* [87, 88]. We predicted 78 624 endolysin sequences across these hosts (e.g. 22 168 in *E. coli*, 20 816 in *Salmonella*). Clustering revealed many viral endolysins with potential activity against multidrug-resistant (MDR) pathogens (Fig. S14), particularly in *Staphylococcus* (*n* = 218), *Escherichia* (*n* = 102), and *Mycobacterium* (*n* = 146). Critically, only 2.3% have been experimentally validated, with most data on *Escherichia* and *Staphylococcus*. Many endolysins, especially those targeting *Klebsiella*, *Mycobacterium*, *Pseudomonas*, *Enterococcus*, and *Acinetobacter*, remain uncharacterized. Distinct low-similarity clusters of *Escherichia*- and *Staphylococcus*-targeting enzymes suggest unique mechanisms. These findings highlight PDVLPD as a promising resource for antimicrobials and underscore the urgent need for experimental validation to harness their clinical potential against antibiotic resistance.

### Diversity of prokaryotic RNA viral lytic proteins

**Figure 7:**
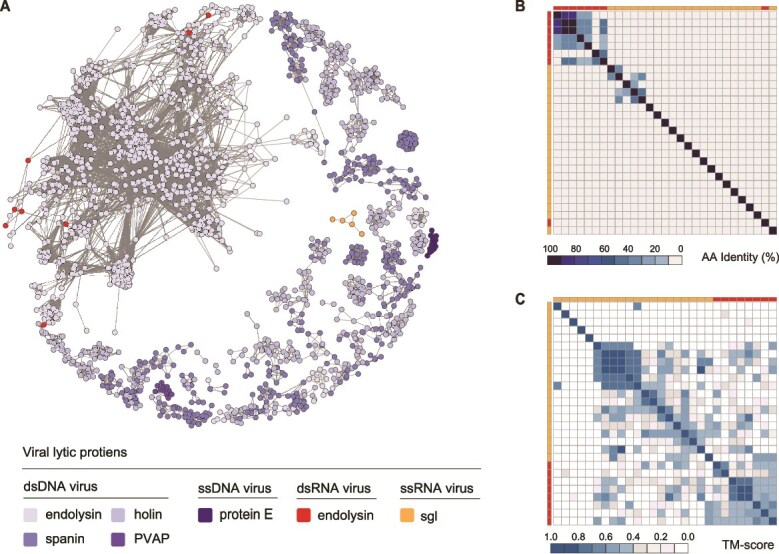
Diversity of prokaryotic RNA viral lytic proteins. (A) Comparison of LyP networks between prokaryotic RNA and DNA viruses. Each node represents a representative protein sequence selected from a sequence cluster. Node colors denote distinct functional or domain categories, whereas edge lengths between nodes reflect the degree of amino acid sequence similarity, with shorter edges indicating higher similarity. For prokaryotic DNA viral endolysins, holins, and spanins, to reduce redundancy, each protein node represents a representative sequence selected from the same sequence cluster. In contrast, due to the limited number of RNA viral LyPs (Pei, PVAP, and pE sequences), all were incorporated into the similarity network without prior clustering. Node colors indicate different functional or domain categories, and the length of connecting edges between nodes reflects amino acid sequence similarity, with shorter edges indicating higher similarity. (B) Sequence similarity matrix of prokaryotic RNA viral LyPs. Representative LyP sequences were clustered using average linkage based on identity values. Color intensity represents the strength of sequence similarity, and the colored bar above indicates the distribution of LyP groups. (C) Structural similarity matrix of prokaryotic RNA viral LyPs. Representative LyP structures were clustered using average linkage based on TM-score values. Color intensity reflects the degree of structural similarity, and the colored bar above represents the distribution of LyP groups. Only LyPs with high predicted confidence (pLDDT ≥70) were included in this analysis.

Having revealed the diversity of prokaryotic DNA viral LyPs, we further analyzed the LyPs from prokaryotic RNA viruses (7 dsRNA, 882 ssRNA). Sequence similarity networks revealed significant divergence from DNA viral LyPs. Among 3263 RNA viral proteins, only eight proteins showed similarity to PDVLPD entries (Fig. 7A). Specifically, the seven dsRNA viruses encoded eight endolysins (*Pseudomonas* phage Φ6 carried two). Five shared 20–40% amino acid similarity with 21 PDVLPD endolysins, all containing glycosidic bond-cleaving domains including transglycosylases, glycosidases, and muramidases. The remaining three contained PET_U40 peptidase domains—a functional endopeptidase domain absent in DNA viruses—suggesting distinct cleavage sites (Fig. S15). Beyond endolysins, no homologs of other DNA viral lysis-associated proteins were detected in dsRNA viruses. In 882 ssRNA viral proteins, only one protein with sequence similarity to known Sgl proteins, whereas structural clustering identified four structural clusters comprising 19 proteins showing structural similarity to known Sgl proteins (Fig. S16, Table S4). Sequence and structural clustering revealed distinct patterns between dsRNA and ssRNA viral-encoded LyPs (Fig. 7B–C): dsRNA virus-encoded endolysins formed three sequence clusters and two structural clusters separate from ssRNA viral LyPs, whereas PET_U40 peptidases formed independent clusters in both analyses. Sgl proteins of ssRNA viruses showed multiple clusters in both analyses, suggesting ongoing differentiation within this group.

## Discussion

Prokaryotic viral LyPs (endolysin, holin, spanin) exhibit substantial sequence diversity but retain structural conservation. Endolysin, a peptidoglycan hydrolase, exhibits conserved substrate-binding structures due to ubiquitous peptidoglycan and specific catalytic requirements [89]. In contrast, holin and spanin show higher sequence variability, reflecting adaptations to diverse host membrane architectures, especially in Gram-positive and Gram-negative bacteria [12, 34]. Their function relies on transmembrane domains or conformational changes rather than conserved catalytic centers like endolysin, explaining their greater diversity [90]. Protein pE, Pei, and PVAP are rare in both the PDVLPD and IMG/VR v4 [74] databases, likely due to their restricted distribution: pE in specific ssDNA viruses [3], Pei targeting archaeal pseudo-peptidoglycan [81, 91], PVAP in acidophilic and thermophilic archaeal viruses [14, 81]. The scarcity of ssDNA and archaeal viral genomes in public databases may further contribute to their underrepresentation.

Building upon our systematic characterization of prokaryotic DNA viral LyP diversity, we found that prokaryotic dsRNA viruses encode endolysins with putative peptidoglycan-degrading activity, including unique catalytic domains (e.g. PET_U40). Concurrently, ssRNA viruses predominantly employ diverse Sgl proteins exhibiting high sequence variability and low conservation [17]. Sequence- and structure-based clustering analyses demonstrate that these RNA viral endolysins share some patterns with their DNA viral counterparts, suggesting shared origins or convergent evolution.

Structural clustering effectively classified LyPs of unknown function, emphasizing structural analysis importance for functional annotation. First, it identified conserved catalytic folds (e.g. GH_24, SLT_related, PET_M15, Ami_2) even among proteins sharing 0% sequence identity, illustrating greater evolutionary stability of structure than sequence in viral LyPs (Fig. S6). Second, it revealed 203 putative LyPs—including endolysins, holins, and spanins—among unannotated hypothetical proteins, particularly from archaeal viruses, revealing a substantial functional reservoir missed by sequence-based methods (Fig. S3). We propose that membrane adaptations are governed by structural diversity, whereas sequence variability reflects distinct eco-evolutionary dynamics. Consequently, unexplored LyP diversity falls into two categories: (i) entirely proteins from understudied groups (e.g. RNA or archaeal viruses), requiring experimental validation, and (ii) uncharacterized structural homologs, requiring systematic comparative and homology studies. Future research should elucidate these undercharacterized proteins, focusing on their functional and evolutionary roles to broaden understanding of viral lysis mechanisms and applications.

The origin and transmission of prokaryotic viral LyPs are fundamental to understanding their diversity and evolution. Numerous cell LyPs exist in bacteria and archaea, functioning in autolysis and physiological regulation [92, 93]. Phylogenetic analyses indicate that viral LyP homologs are prevalent in prokaryotic genomes, suggesting ancient HGT among viruses, bacteria, and archaea. Evolutionary origins differ markedly: endolysin and holin homologs potential Last Universal Common Ancestor (LUCA) origins, whereas spanin are restricted to Gram-negative bacteria, suggesting lineage-specific evolution (though independent viral origins remain possible). Despite challenges in tracing ancient HGT, we identified multiple recent HGT events. Gene flow predominantly occurs from bacteria to viruses, supporting bacteriophage exaptation of bacterial genes for adaptability [94]. These genes—encoding peptidoglycan hydrolases (e.g. lysozymes, endopeptidases)—originate from bacterial processes like cell wall remodeling and sporulation [95]. Phages repurpose these mechanisms for host lysis post-transfer. Some HGT directionality may require reevaluation due to phylogenetic rooting uncertainties. Additionally, HGT often involves modular units, not single genes, driving viral lytic system evolution. These findings highlight complex virus-prokaryote evolutionary interplay, shaping LyP diversity and adaptation mechanisms.

Prokaryotic viral lysis system complexity correlates with genome size: larger genomes (> 140 kb) encode more diverse lytic systems to breach robust host barriers and accommodate bulkier capsids [96], whereas smaller (20–80 kb) genomes exhibit simpler lysis system (e.g. m-EAD endolysin, 4-TMRs holin, and USP spanin) with higher gene compactness. m-EAD distribution suggests that domain recombination and fusion via non-homologous recombination and HGT drive multi-catalytic-domain LyPs evolution. Host specificity further shapes composition: Gram-positive-targeting phages predominantly encode amidase- and endopeptidase-type endolysin, whereas Gram-negative-targeting phages feature muramidase- and transglycosylase-type endolysins and spanins (absent in Gram-positive systems). Additionally, *Cyanobacteria* (Gram-negative) lack typical outer membrane porins [97], potentially preventing spanin function and driving alternative strategies. Endolysins and holins universally target cell wall and cytoplasmic membrane, respectively [90], forming an efficient synergistic module that expands host range. Collectively, genome size, host envelope complexity, and capsid dimensions probably drive viral lysis system adaptation.

The antibiotic resistance crisis causes over 700 000 deaths annually [87, 98]. Conventional antibiotics are increasingly ineffective against MDR bacteria, highlighting the urgent need for alternative treatments. Bacteriophage-derived endolysins have emerged as agents offering potent bactericidal activity, target specificity, and low resistance development [99]. Tools such as DeepMineLys and VersaTile have accelerated endolysin research. Whereas this study focuses on WHO-priority MDR pathogens [88], our analysis highlights the vast therapeutic potential of bacteriophage endolysin against these pathogens, though experimental validation remains limited and concentrated on *Escherichia* and *Staphylococcus*. By constructing a comprehensive LyP database, this study expands endolysin exploration and provides key insights for enzyme engineering, and clinical translation toward next-generation antimicrobial therapeutics.

## Supplementary Material

Suppl_Info-PDVLPD-20250828-clean_wraf200

Supplementary_Table_1_wraf200

Supplementary_Table_2_wraf200

Supplementary_Table_3_wraf200

Supplementary_Table_4_wraf200

Supplementary_Table_5_wraf200

## Data Availability

All data used in this study were obtained from publicly available databases. Endolysin sequences were sourced from Phalp [22], holin sequences from TCDB [26], and spanin sequences from SpaninDB [12]. Pei, pE, and PVAP sequences were collected from the NCBI RefSeq database (released on July 2023) [27], Prokaryotic viral data were obtained from ICTV (VMR_MSL38_v2) [30]. Complete and chromosomal-level bacterial and archaeal genome data were retrieved from NCBI GenBank (downloaded on July 25, 2023) [100]. Uncultured viral genome sequences were sourced from IMG/VR v4 (high-confidence genomes only, released on 20 September 2022) [74]. All data are provided as a publicly deposited dataset on Figshare (DOI: 10.6084/m9.figshare.28425179). Although this resource does not currently support interactive queries, all files are fully accessible for analysis. This paper did not generate any new unique reagents or any new code.
