# Supplementary material for: Diversity and evolution of prokaryotic viral lytic proteins
Source: ISME J. 2025 Oct 8;19(1):wraf200. doi: 10.1093/ismejo/wraf200 (PMC12596118; doi:10.1093/ismejo/wraf200)
Supplement: Suppl_Info-PDVLPD-20250828-clean_wraf200 [file suppl_info-pdvlpd-20250828-clean_wraf200.pdf]

Supplementary Materials for

Diversity and Evolution of Prokaryotic Viral Lytic Proteins

Running title: Lytic Proteins Diversity & Evolution

Ting Yang[1, 2], Mujie Zhang[1, 2], Yi Yi[1], Yecheng Wang[1], Zhiwei Wang[1], Rui Zhang[3], Xiang Xiao[1, 2], Huahua Jian[1, 2]*

[1]State Key Laboratory of Microbial Metabolism, Joint International Research Laboratory of Metabolic & Development Sciences, School of Life Sciences & Biotechnology, Shanghai Jiao Tong University, 200240, Shanghai, PR China

[2]Yazhou Bay Institute of Deepsea Sci-Tech, Shanghai Jiao Tong University, 572025, Sanya, Hainan, PR China

[3]Archaeal Biology Center, Synthetic Biology Research Center, Shenzhen Key Laboratory of Marine Microbiome Engineering, Key Laboratory of Marine Microbiome Engineering of Guangdong Higher Education Institutes, Institute for Advanced Study, Shenzhen University, 518055, Shenzhen, PR China

*Correspondence author: jiandy@sjtu.edu.cn

Huahua Jian

School of Life Sciences & Biotechnology, Shanghai Jiao Tong University

No. 800 Dongchuan Road, Minhang District, 200240, PR China

This PDF file includes:

Figs. S1 to S16

Tables S1 to S5

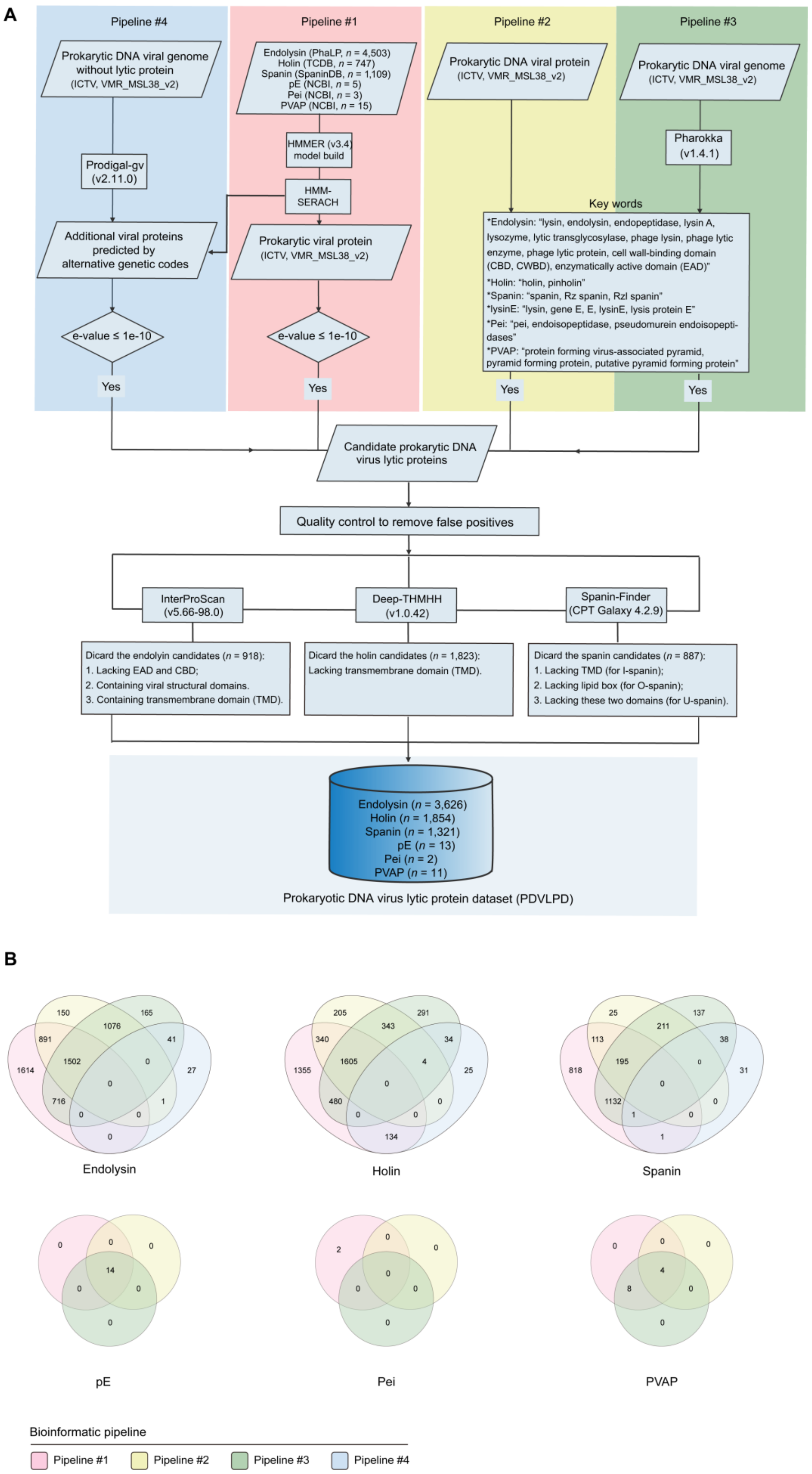
A
Pipeline #4
Prokarytic DNA viral genome without lytic protein (ICTV, VMR_MSL38_v2)
Prodigal-gv (v2.11.0)
Additional viral proteins predicted by alternative genetic codes
e-value ≤ 1e-10
Yes
Pipeline #1
Endolysin (PhaLP, n = 4,503)
Holin (TCDB, n = 747)
Spanin (SpaninDB, n = 1,109)
pE (NCBI, n = 5)
Pei (NCBI, n = 3)
PVAP (NCBI, n = 15)
HMMER (v3.4) model build
HMM-SERACH
Prokaryotic viral protein (ICTV, VMR_MSL38_v2)
e-value ≤ 1e-10
Yes
Pipeline #2
Prokarytic DNA viral protein (ICTV, VMR_MSL38_v2)
Yes
Pipeline #3
Prokarytic DNA viral genome (ICTV, VMR_MSL38_v2)
Pharokka (v1.4.1)
Key words
*Endolysin: "lysin, endolysin, endopeptidase, lysin A, lysozyme, lytic transglycosylase, phage lysin, phage lytic enzyme, phage lytic protein, cell wall-binding domain (CBD, CWBD), enzymatically active domain (EAD)"
*Holin: "holin, pinholin"
*Spanin: "spanin, Rz spanin, Rzl spanin"
*lysinE: "lysin, gene E, E, lysinE, lysis protein E"
*Pei: "pei, endoisopeptidase, pseudomurein endoisopepti-dases"
*PVAP: "protein forming virus-associated pyramid, pyramid forming protein, putative pyramid forming protein"
Yes
Candidate prokarytic DNA virus lytic proteins
Quality control to remove false positives
InterProScan (v5.66-98.0)
Deep-THMHH (v1.0.42)
Spanin-Finder (CPT Galaxy 4.2.9)
Discard the endolyin candidates (n = 918):
1. Lacking EAD and CBD;
2. Containing viral structural domains.
3. Containing transmembrane domain (TMD).
Dicard the holin candidates (n = 1,823):
Lacking transmembrane domain (TMD).
Dicard the spanin candidates (n = 887):
1. Lacking TMD (for I-spanin);
2. Lacking lipid box (for O-spanin);
3. Lacking these two domains (for U-spanin).
Endolysin (n = 3,626)
Holin (n = 1,854)
Spanin (n = 1,321)
pE (n = 13)
Pei (n = 2)
PVAP (n = 11)
Prokaryotic DNA virus lytic protein dataset (PDVLPD)
B
Endolysin
Holin
Spanin
pE
Pei
PVAP
Bioinformatic pipeline
Pipeline #1
Pipeline #2
Pipeline #3
Pipeline #4

Fig. S1. Construction workflow of the PDVLPD. (A) Overview of the four bioinformatics pipelines (Pipeline #1–4) and quality control steps employed for the assembly of the PDVLPD. Pipeline #1 utilizes HMMER [1] to build Hidden Markov Models (HMM) based on known LyPs from public databases, which are subsequently applied to screen viral proteins from the ICTV database [2] for LyP sequences. Pipeline #2 extracts relevant sequences from ICTV viral proteins through keyword-based searches. Pipeline #3 employs Pharokka [3] to re-annotate ICTV viral genomes, enabling the identification of additional viral LyPs. Pipeline #4 applies the virus-specific Prodigal-gv [4] algorithm to predict potential LyP sequences from viral genomes lacking prior LyP annotations. These pipelines collectively generate a candidate LyP library, which undergoes rigorous quality control to eliminate false positives, resulting in a high-quality prokaryotic virus LyP dataset. **(B)** Contribution of different bioinformatics pipelines to LyP identification. Venn diagrams illustrate the distribution of LyP categories (endolysin, holin, spanin, pE, Pei, and PVAP) identified by each pipeline. Non-overlapping regions represent LyPs uniquely detected by a single pipeline, underscoring the importance of integrating multiple bioinformatics approaches for comprehensive LyP discovery.

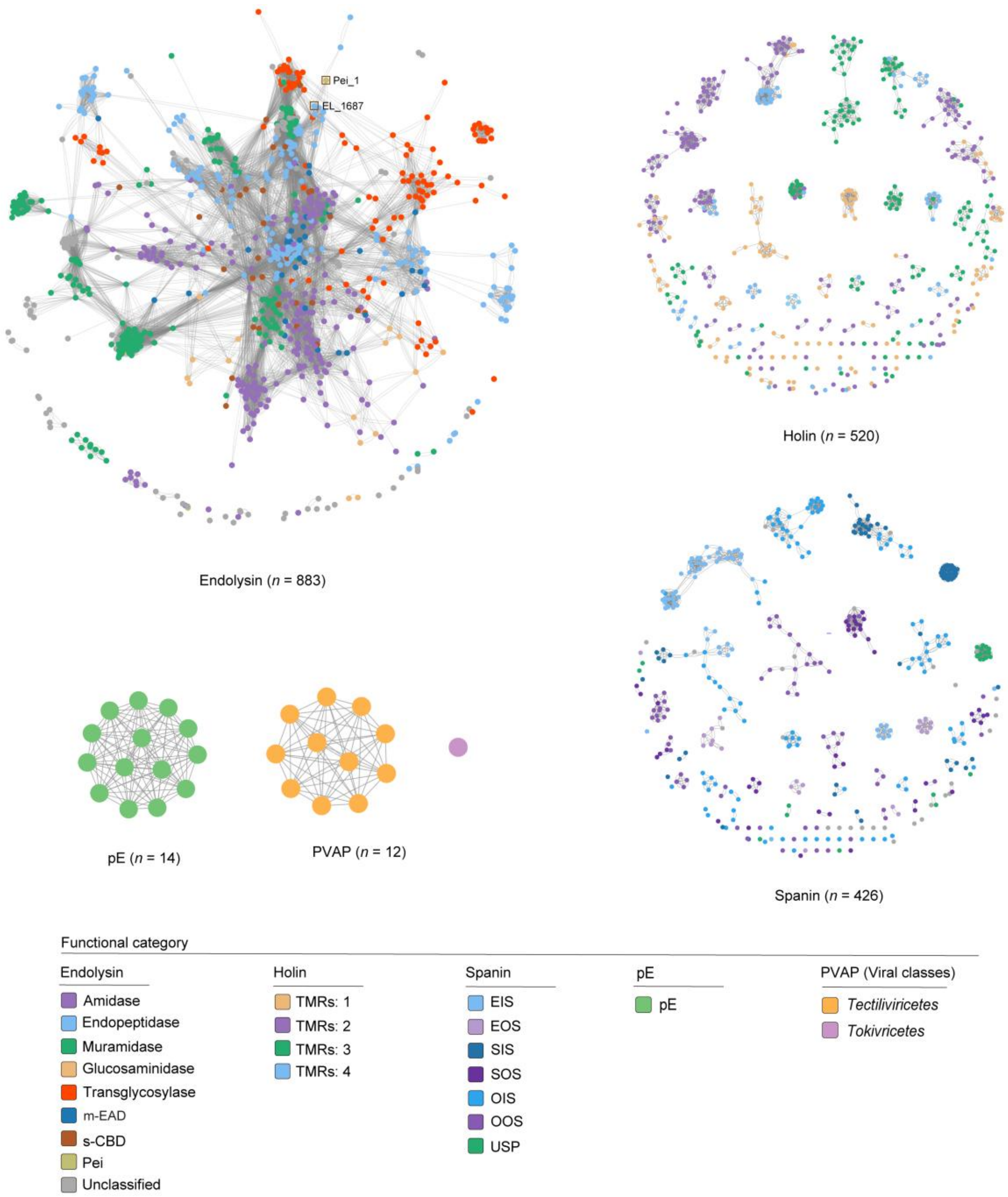

Fig. S2. Sequence similarity network of lytic proteins in the PDVLPD. Each node represents a representative protein sequence selected from a sequence cluster. Node colors denote distinct functional or domain categories, whereas edge lengths between nodes reflect the degree of amino acid sequence similarity, with shorter edges indicating higher similarity. For endolysin, holin, and spanin, clustering was performed to reduce redundancy, and a representative sequence from each cluster was selected for network construction. In contrast, due to the limited number of Pei, PVAP, and pE sequences, all proteins within these categories were directly

incorporated into the similarity network without prior clustering. This approach ensures comprehensive representation of both abundant and rare LyP families.

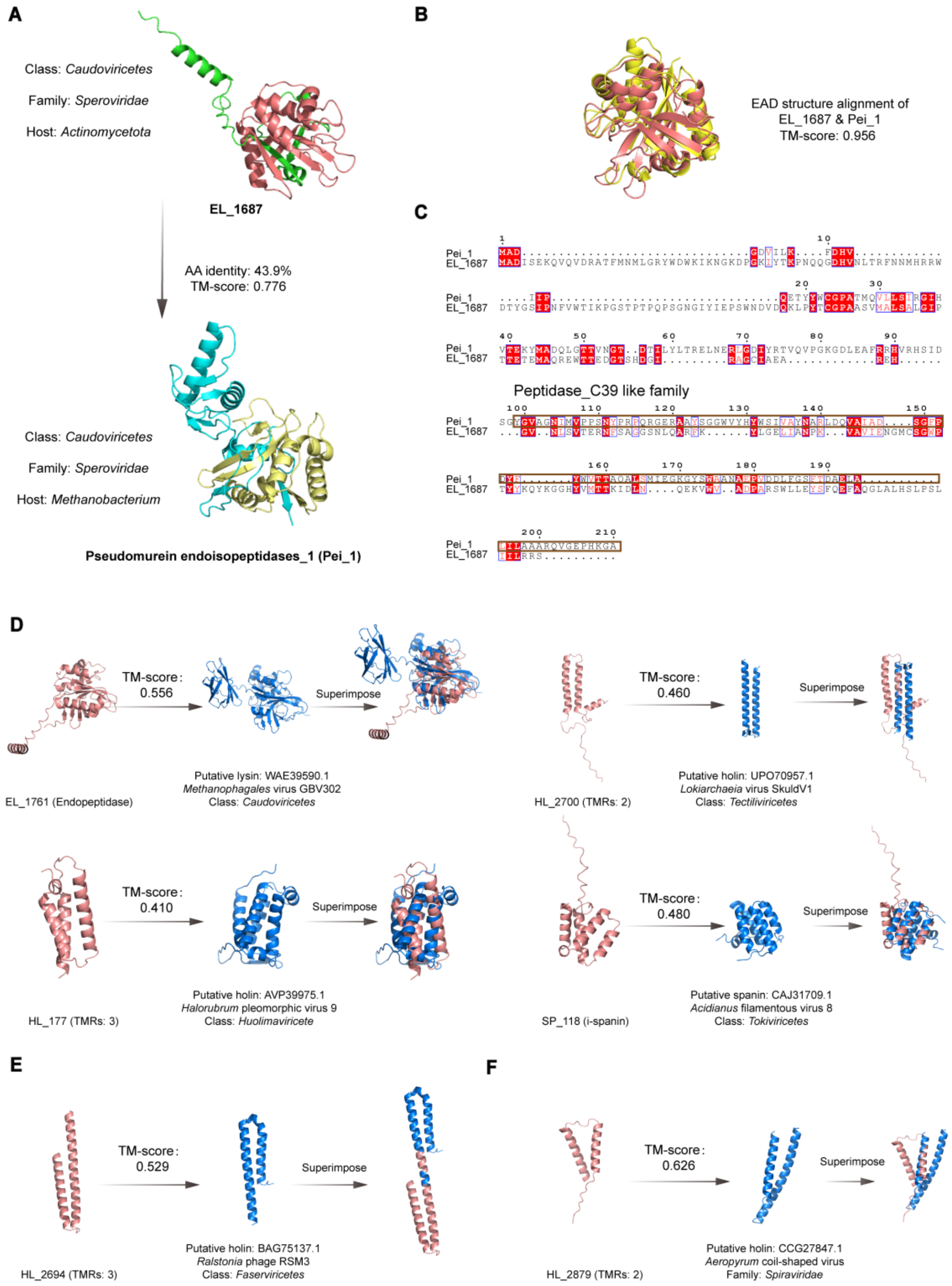

Fig. S3. Comparative analysis of sequence and structural similarities between the lytic proteins of archaeal virus and bacteriophage. (A and B) Structural alignment of the Pei_1 protein from the *Methanobacterium* virus C158 with the bacteriophage protein Endolysin_1687, illustrating both the full-length protein (A) and the enzymatically active domain (EAD) (B). **(C)** Amino acid sequence alignment of Pei_1

and Endolysin_1687, with identical residues highlighted in red and the conserved peptidase C39-like domain delineated by a black box. **(D-F)** Structural comparison of LyPs from diverse archaeal viruses with those from bacteriophages, demonstrating conserved structural motifs and functional domains across viral lineages. This comparative analysis highlights evolutionary and functional parallels between archaeal and bacterial virus lytic systems.

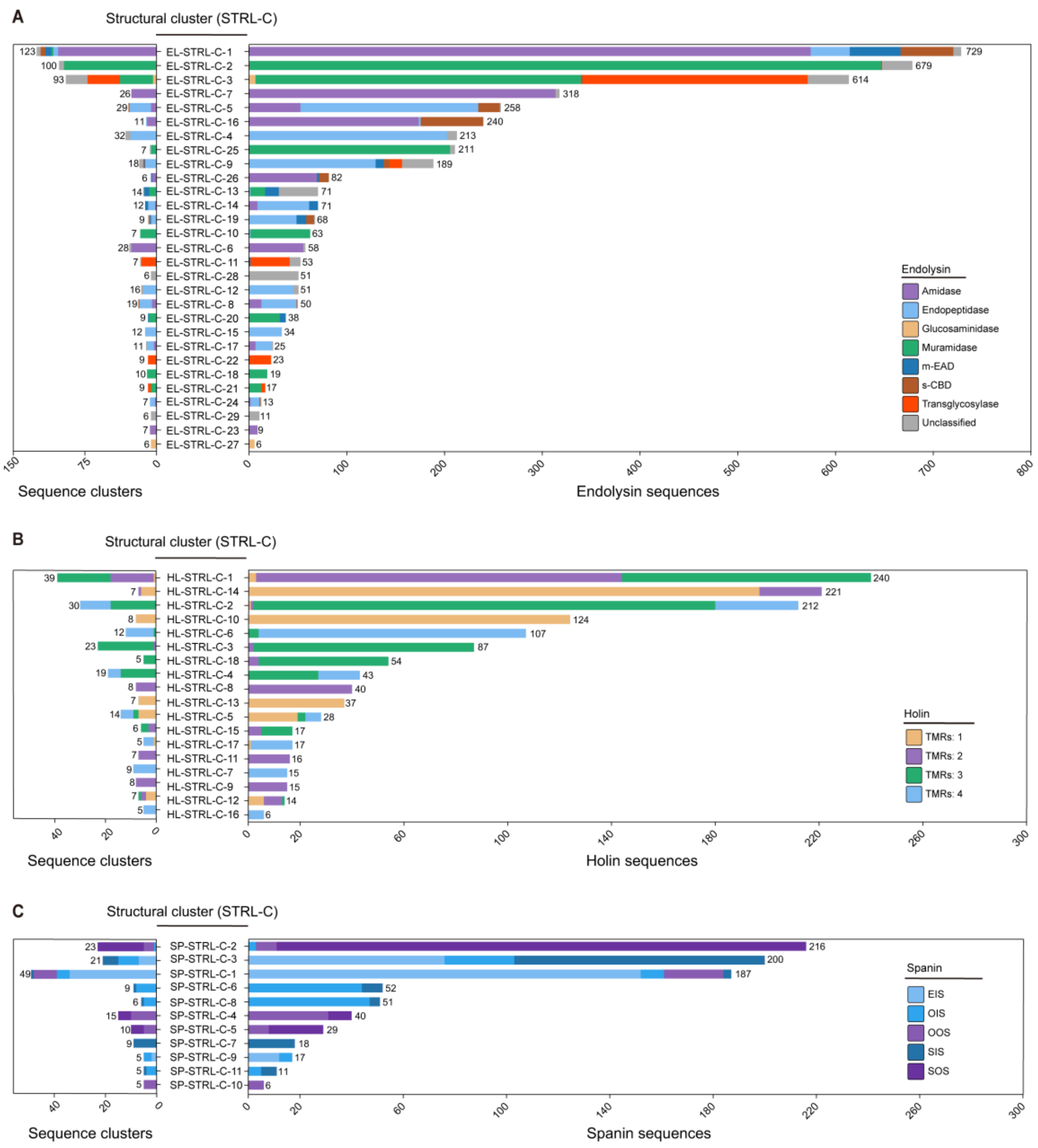

Fig. S4. Quantitative relationships between sequence clusters and structural clusters of prokaryotic viral lytic proteins, showing member counts and functional composition distributions. (A) Endolysin sequence versus structural clusters with member counts and functional mechanism proportions; **(B)** Holin sequence versus structural clusters with member counts and functional mechanism proportions; **(C)** Spanin sequence versus structural clusters with member counts and functional mechanism proportions. In each panel, the upper bar plot displays the number of sequences contained within each structural cluster, whereas the lower bar

plot shows the corresponding number of sequence clusters. Only clusters containing ≥5 members are displayed for comparative analysis.

A

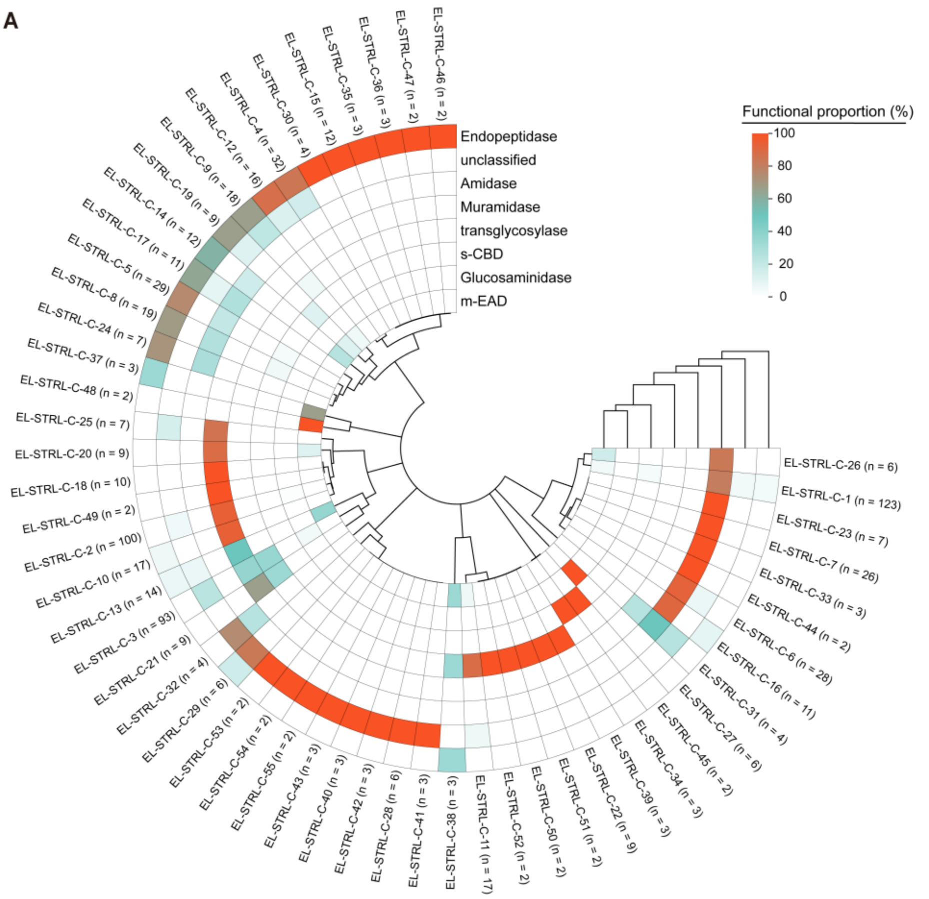
Functional proportion (%)
100
80
60
40
20
0
Endopeptidase
unclassified
Amidase
Muramidase
transglycosylase
s-CBD
Glucosaminidase
m-EAD
EL-STRL-C-46 (n = 2)
EL-STRL-C-47 (n = 2)
EL-STRL-C-36 (n = 3)
EL-STRL-C-35 (n = 3)
EL-STRL-C-15 (n = 12)
EL-STRL-C-30 (n = 4)
EL-STRL-C-4 (n = 32)
EL-STRL-C-12 (n = 16)
EL-STRL-C-9 (n = 18)
EL-STRL-C-19 (n = 9)
EL-STRL-C-14 (n = 12)
EL-STRL-C-17 (n = 11)
EL-STRL-C-5 (n = 29)
EL-STRL-C-8 (n = 19)
EL-STRL-C-24 (n = 7)
EL-STRL-C-37 (n = 3)
EL-STRL-C-48 (n = 2)
EL-STRL-C-25 (n = 7)
EL-STRL-C-20 (n = 9)
EL-STRL-C-18 (n = 10)
EL-STRL-C-49 (n = 2)
EL-STRL-C-2 (n = 100)
EL-STRL-C-10 (n = 17)
EL-STRL-C-13 (n = 14)
EL-STRL-C-3 (n = 93)
EL-STRL-C-21 (n = 9)
EL-STRL-C-32 (n = 4)
EL-STRL-C-29 (n = 6)
EL-STRL-C-53 (n = 2)
EL-STRL-C-54 (n = 2)
EL-STRL-C-55 (n = 2)
EL-STRL-C-43 (n = 3)
EL-STRL-C-40 (n = 3)
EL-STRL-C-42 (n = 3)
EL-STRL-C-28 (n = 6)
EL-STRL-C-41 (n = 3)
EL-STRL-C-38 (n = 3)
EL-STRL-C-11 (n = 17)
EL-STRL-C-52 (n = 2)
EL-STRL-C-50 (n = 2)
EL-STRL-C-51 (n = 2)
EL-STRL-C-22 (n = 9)
EL-STRL-C-39 (n = 3)
EL-STRL-C-34 (n = 3)
EL-STRL-C-45 (n = 2)
EL-STRL-C-27 (n = 6)
EL-STRL-C-31 (n = 4)
EL-STRL-C-16 (n = 11)
EL-STRL-C-6 (n = 28)
EL-STRL-C-44 (n = 2)
EL-STRL-C-33 (n = 3)
EL-STRL-C-7 (n = 26)
EL-STRL-C-23 (n = 7)
EL-STRL-C-1 (n = 123)
EL-STRL-C-26 (n = 6)

B

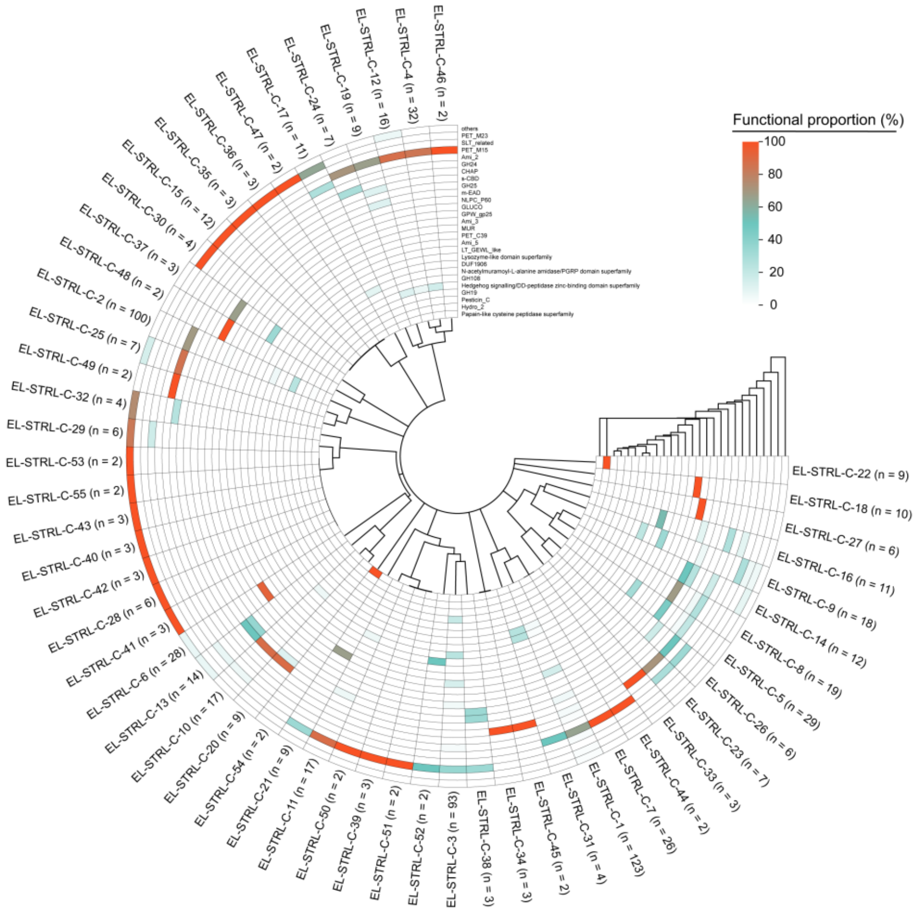
Functional proportion (%)
100
80
60
40
20
0
others
PET_M23
SLT_related
PET_M15
Ami_2
GH24
CHAP
s-CBD
GH25
m-EAD
NLPC_P60
GLUCO
GPW_gp25
Ami_3
MUR
PET_C39
Ami_5
LT_GEWL_like
Lysozyme-like domain superfamily
DUF1906
N-acetylmuramoyl-L-alanine amidase/PGRP domain superfamily
GH108
Hedgehog signalling/DD-peptidase zinc-binding domain superfamily
GH19
Pesticin_C
Hydro_2
Papain-like cysteine peptidase superfamily
EL-STRL-C-46 (n = 2)
EL-STRL-C-4 (n = 32)
EL-STRL-C-12 (n = 16)
EL-STRL-C-19 (n = 9)
EL-STRL-C-24 (n = 7)
EL-STRL-C-17 (n = 11)
EL-STRL-C-47 (n = 2)
EL-STRL-C-36 (n = 3)
EL-STRL-C-35 (n = 3)
EL-STRL-C-15 (n = 12)
EL-STRL-C-30 (n = 4)
EL-STRL-C-37 (n = 3)
EL-STRL-C-48 (n = 2)
EL-STRL-C-2 (n = 100)
EL-STRL-C-25 (n = 7)
EL-STRL-C-49 (n = 2)
EL-STRL-C-32 (n = 4)
EL-STRL-C-29 (n = 6)
EL-STRL-C-53 (n = 2)
EL-STRL-C-55 (n = 2)
EL-STRL-C-43 (n = 3)
EL-STRL-C-40 (n = 3)
EL-STRL-C-42 (n = 3)
EL-STRL-C-28 (n = 6)
EL-STRL-C-41 (n = 3)
EL-STRL-C-6 (n = 28)
EL-STRL-C-13 (n = 14)
EL-STRL-C-10 (n = 17)
EL-STRL-C-20 (n = 9)
EL-STRL-C-54 (n = 2)
EL-STRL-C-21 (n = 9)
EL-STRL-C-11 (n = 17)
EL-STRL-C-50 (n = 2)
EL-STRL-C-39 (n = 3)
EL-STRL-C-51 (n = 2)
EL-STRL-C-52 (n = 2)
EL-STRL-C-3 (n = 93)
EL-STRL-C-38 (n = 3)
EL-STRL-C-34 (n = 3)
EL-STRL-C-45 (n = 2)
EL-STRL-C-31 (n = 4)
EL-STRL-C-1 (n = 123)
EL-STRL-C-7 (n = 26)
EL-STRL-C-44 (n = 2)
EL-STRL-C-33 (n = 3)
EL-STRL-C-23 (n = 7)
EL-STRL-C-26 (n = 6)
EL-STRL-C-5 (n = 29)
EL-STRL-C-8 (n = 19)
EL-STRL-C-14 (n = 12)
EL-STRL-C-9 (n = 18)
EL-STRL-C-16 (n = 11)
EL-STRL-C-27 (n = 6)
EL-STRL-C-18 (n = 10)
EL-STRL-C-22 (n = 9)

Fig. S5. Structural-functional correlations of endolysins. (A) Distribution of endolysins with different catalytic mechanisms across endolysin structural clusters. **(B)** Distribution of catalytic domains (EADs) from endolysins with distinct mechanisms within endolysin structural clusters. Structural clustering was performed on 779 representative endolysin structures using the easy-cluster module in Foldseek (v1.3) [5] with stringent parameters (alignment coverage ≥ 70%, TM-score ≥ 0.4, and E-value < 0.001). Heatmaps depict the proportional representation of endolysins or endolysin domains within specific structural clusters. Only clusters containing endolysins with high-confidence predicted structures (pLDDT ≥ 70) are displayed for clarity and reliability.

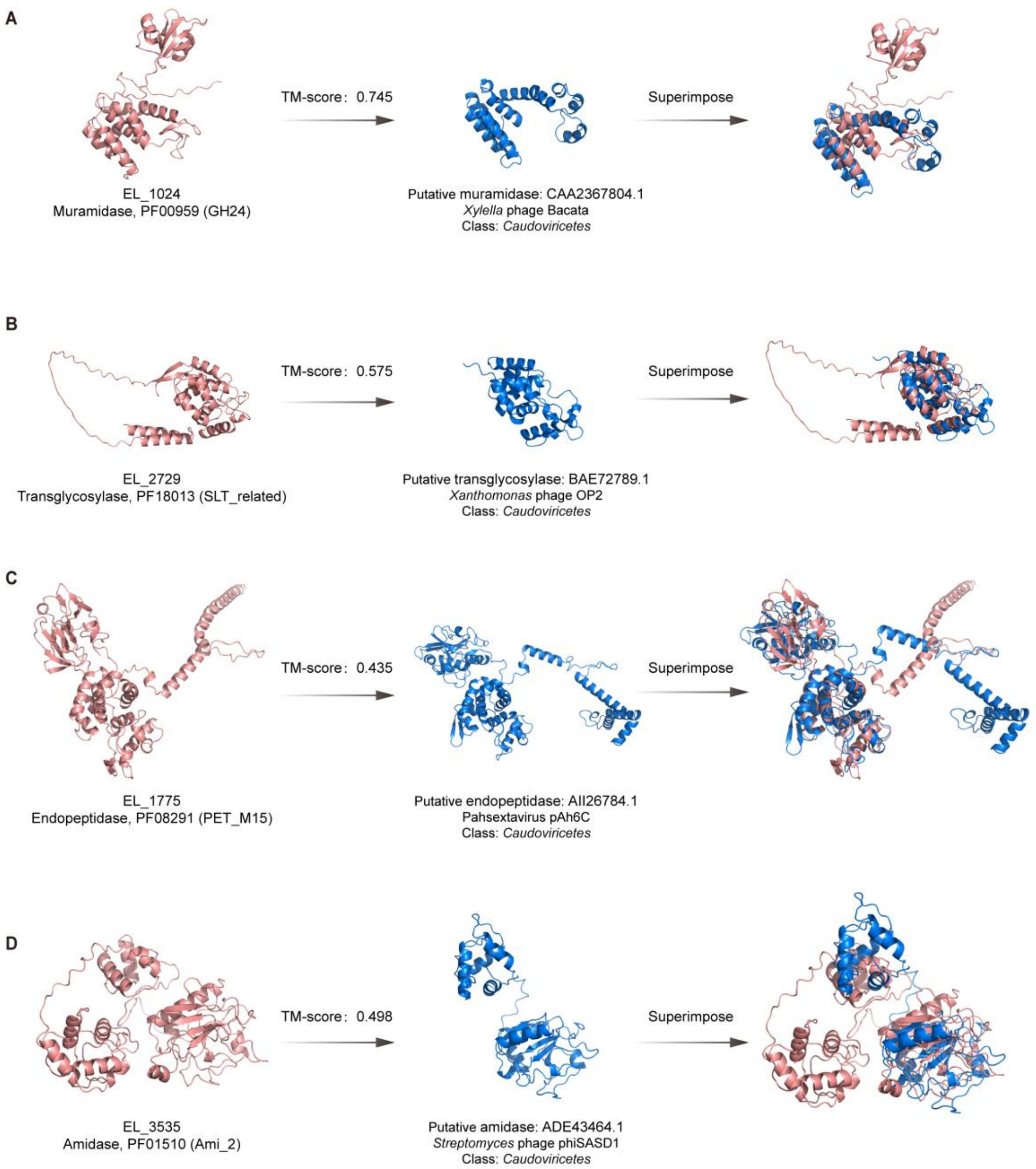

Fig. S6. Functional prediction of uncharacterized lytic proteins based on structural comparisons. (A) Structural alignment of CAA2367804.1 with a known muramidase. **(B)** Structural alignment of BAE72789.1 with a known transglycosylase. **(C)** Structural alignment of AII26784.1 with a known endopeptidase. **(D)** Structural alignment of ADE43464.1 with a known amidase. TM-scores were calculated using Foldseek (v1.3) [5] easy-search mode.

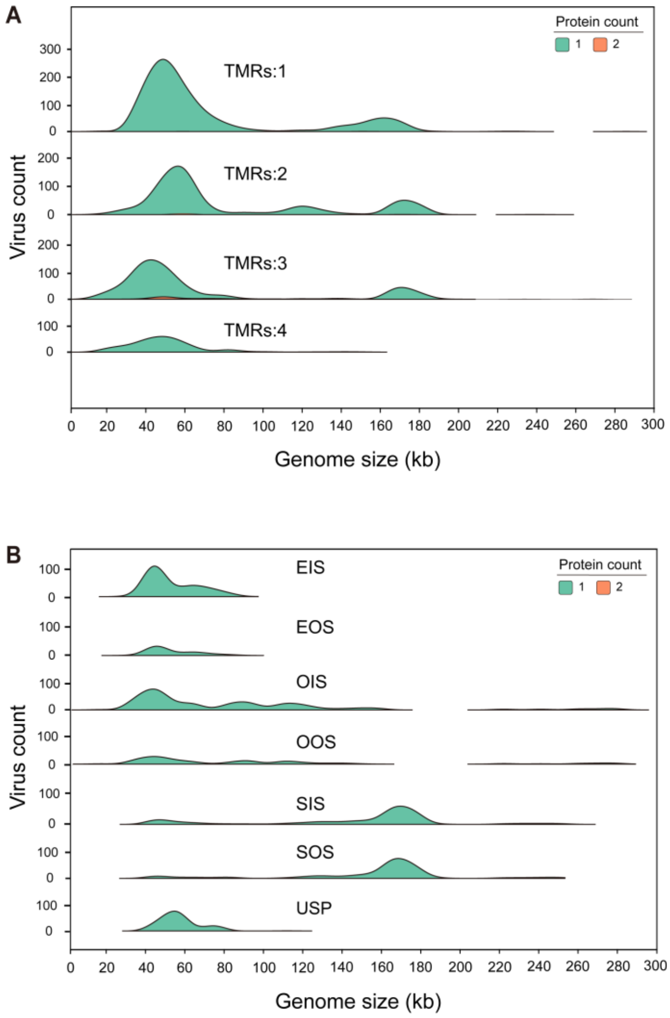

Fig. S7. Distribution patterns of holin and spanin relative to viral genome size. **(A)** Distribution patterns of holin categorized by the number of transmembrane regions (TMRs: 1–4) in relation to viral genome size. **(B)** Distribution patterns of spanin classified into different types based on their membrane topology: SOS (Separated outer membrane spanin), SIS (Separated inner membrane spanin), EOS (Embedded outer membrane spanin), EIS (Embedded iInner membrane spanin), OOS (Overlapped outer membrane spanin), OIS (Overlapped inner membrane spanin), and USP (Unimolecular spanin). Green and orange bars represent the copy numbers of LyPs within a single viral genome, indicating one and two copies, respectively.

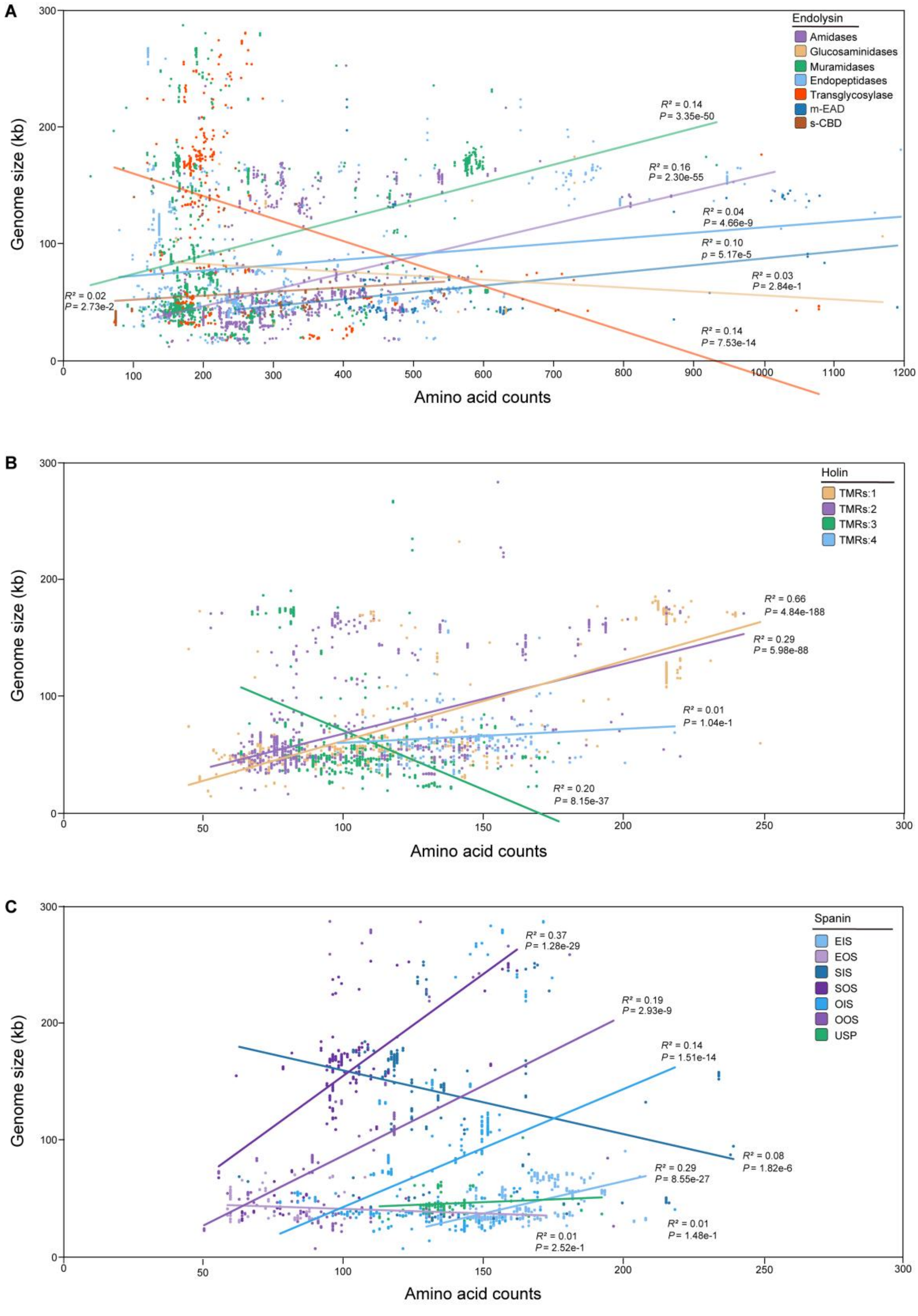

Fig. S8. Correlation analysis between amino acid count of lytic proteins and viral genome size. (A) Correlation between amino acid count and viral genome size for different functional types of endolysin. Data points, color-coded by endolysin type,

represent distinct functional groups. Regression lines with determination coefficient (R^2) and significance level (*P* value). Abbreviations: s-CBD (sole cell wall binding domain), m-EAD (multiple enzymatic activity domain). **(B)** Correlation between amino acid count of holin proteins, stratified by transmembrane region (TMR) numbers (1–4), and viral genome size. Data points in distinct colors indicate holins with varying TMR counts. (C) Correlation between amino acid count and viral genome size for different spanin categories. Data points, distinguished by color, represent spanin types: SOS (Separated outer membrane spanin), SIS (Separated inner membrane spanin), EOS (Embedded outer membrane spanin), EIS (Embedded iInner membrane spanin), OOS (Overlapped outer membrane spanin), OIS (Overlapped inner membrane spanin), and USP (Unimolecular spanin). Pearson's *r* was used for correlation analysis.

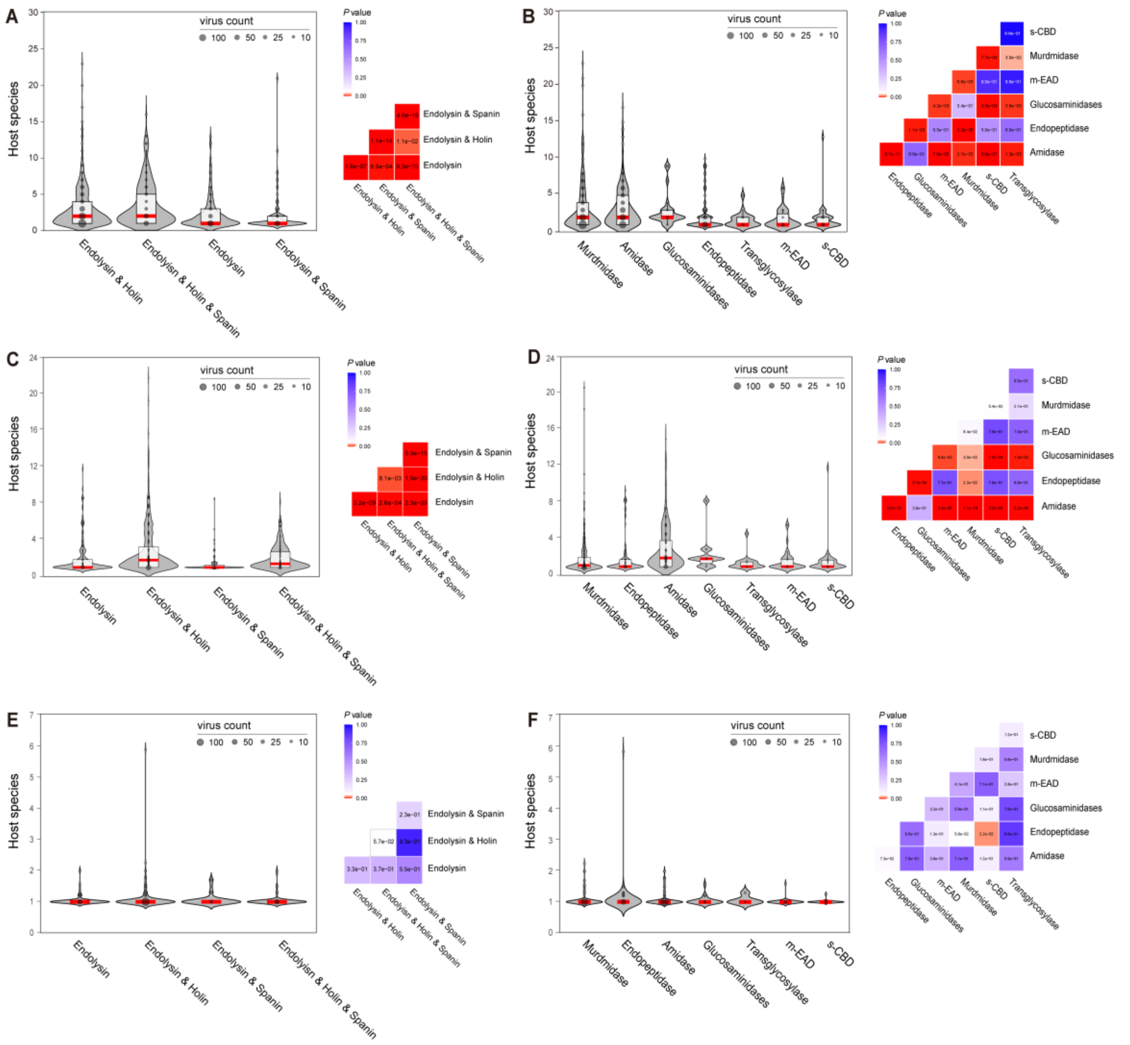

Fig. S9. Comparison of host ranges in prokaryotic dsDNA viruses with different lysis systems and functional endolysins. (A) Host range comparison among prokaryotic dsDNA viruses with distinct lysis systems. **(B)** Host range comparison among prokaryotic dsDNA viruses with different functional endolysin types. **(C)** Host range comparison among prokaryotic dsDNA viruses with distinct lysis systems at the phylum level under random sampling of 2000 species per phylum. **(D)** Host range comparison among prokaryotic dsDNA viruses with different functional endolysin types at the phylum level under random sampling of 2000 species per phylum. **(E)** Host range comparison among prokaryotic dsDNA viruses with distinct lysis systems at the phylum level under random sampling of 100 species per phylum. **(F)** Host range comparison among prokaryotic dsDNA viruses with different functional endolysin types at the phylum level under random sampling of 100 species per

phylum. Statistical significance was evaluated using the Wilcoxon test, with *P* values shown in the heatmap adjacent to the violin plots. The box in each violin plot represents the interquartile range (IQR), the red central line indicates the median, and whiskers extend to 1.5×IQR. Circle size is proportional to the number of viral genomes represented.

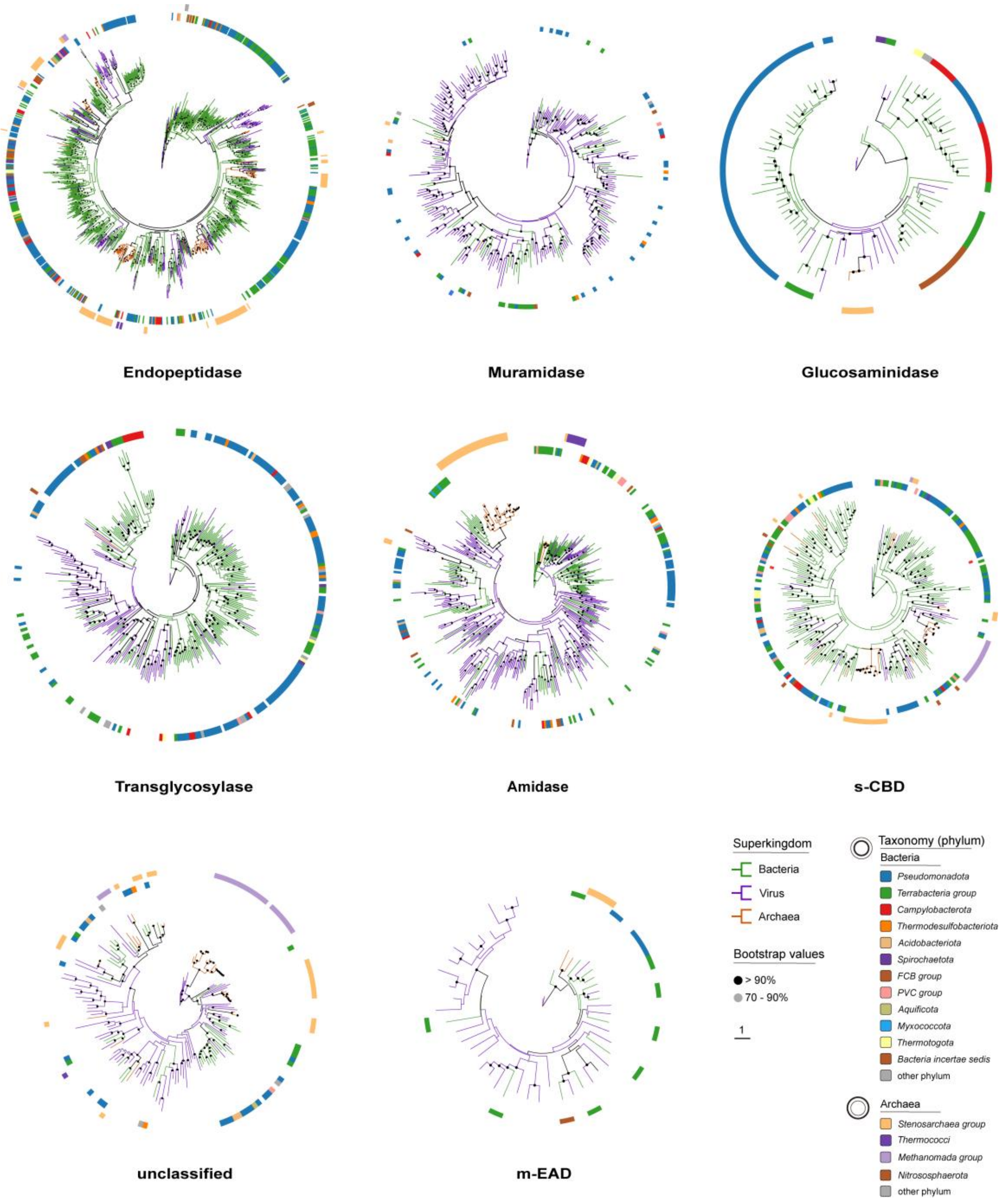

Fig. S10. Phylogenetic analysis of endolysins categorized by functional type. Phylogenetic trees were constructed using the MFP model in IQ-TREE (v2.13) [6], with branch support assessed through 1,000 bootstrap replicates. Scale bar indicates 1 amino acid substitution per site. Tree visualization and annotation were performed using Chiplot (https://www.chiplot.online/) [7]. To manage the extensive number of bacterial homologous LyPs, TaxonKit (v0.18.0) [8] was employed to conduct taxonomic-level sampling, ensuring a structured phylogenetic representation and

functional distribution. Branches in the phylogenetic trees are color-coded to indicate the domain of origin of the LyPs (viruses, bacteria, archaea). The outer circular rings (from outer to inner) annotate archaeal and bacterial taxonomic classifications at the phylum level. Node colors represent bootstrap support values, with nodes exceeding 90% bootstrap support highlighted in black. Functional annotations include s-CBD (sole cell wall binding domain) and m-EAD (multiple enzymatic activity domains), providing insights into the evolutionary and functional diversity of endolysins.

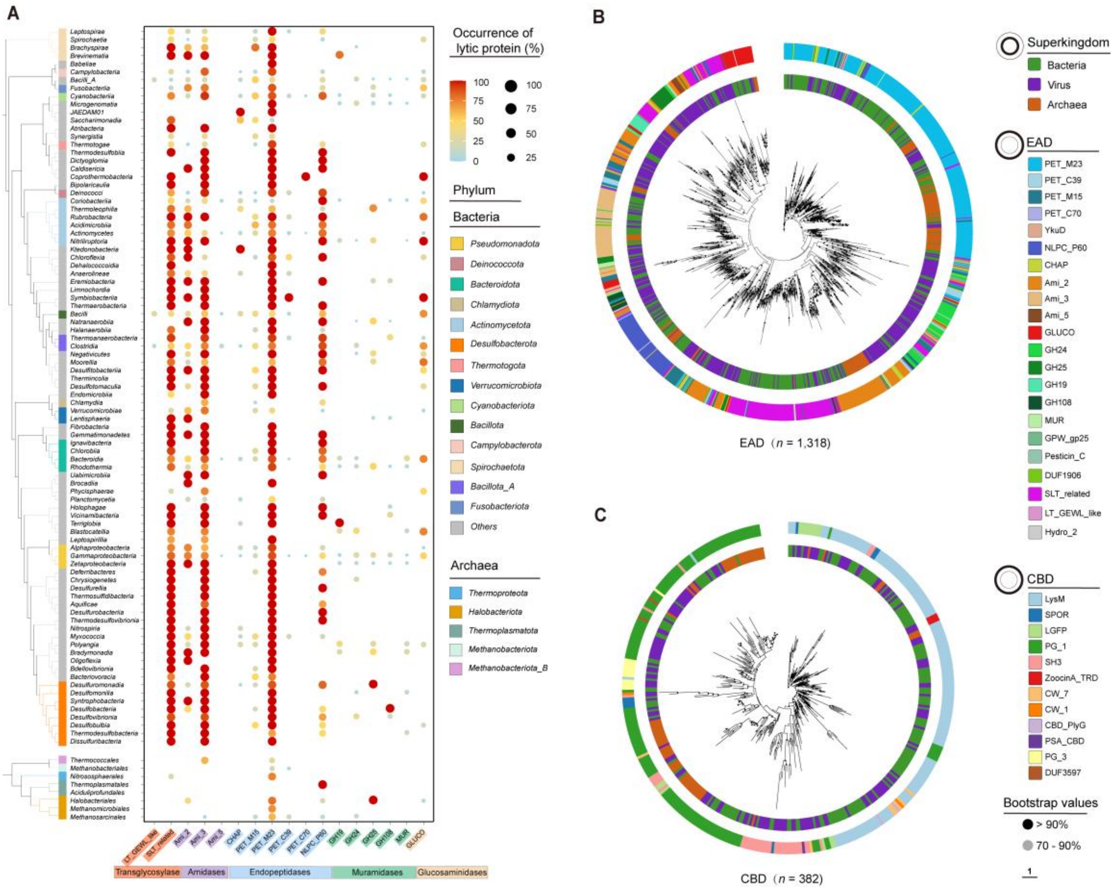

Fig. S11. Distribution and phylogeny of DNA virus lytic protein domains. (A) Distribution of DNA virus LyP domains across bacteria (classified by family) and archaea (classified by phylum). The reference phylogenetic tree was obtained from the GTDB database (release 220) [9], where rectangle colors represent phylum-level taxonomy, and bacterial phyla containing fewer than 20 genomes are shown in gray. The occurrence of endolysin proteins is quantitatively displayed through the color and size of circles. **(B)** Phylogenetic tree of Endolysin-EADs, comprising 592 viral, 548 bacterial, and 178 archaeal EAD amino acid sequences. **(C)** Phylogenetic tree of Endolysin-CBDs, including 149 viral, 168 bacterial, and 65 archaeal CBD amino acid sequences. All phylogenetic trees were constructed using the MFP model in IQ-TREE (v2.13) [6] with 1,000 bootstrap replicates to assess branch support. Scale bar indicates 1 amino acid substitution per site. Tree visualization and annotation were performed using Chiplot [7]. Branches are color-coded to indicate the source of LyP domains (viral, bacterial, archaeal). The outer rings of the phylogenetic trees display

taxonomic information (for viruses, bacteria, and archaea) and the types of Endolysin EADs and CBDs. Node colors represent bootstrap support values, with nodes exceeding 90% support highlighted in black.

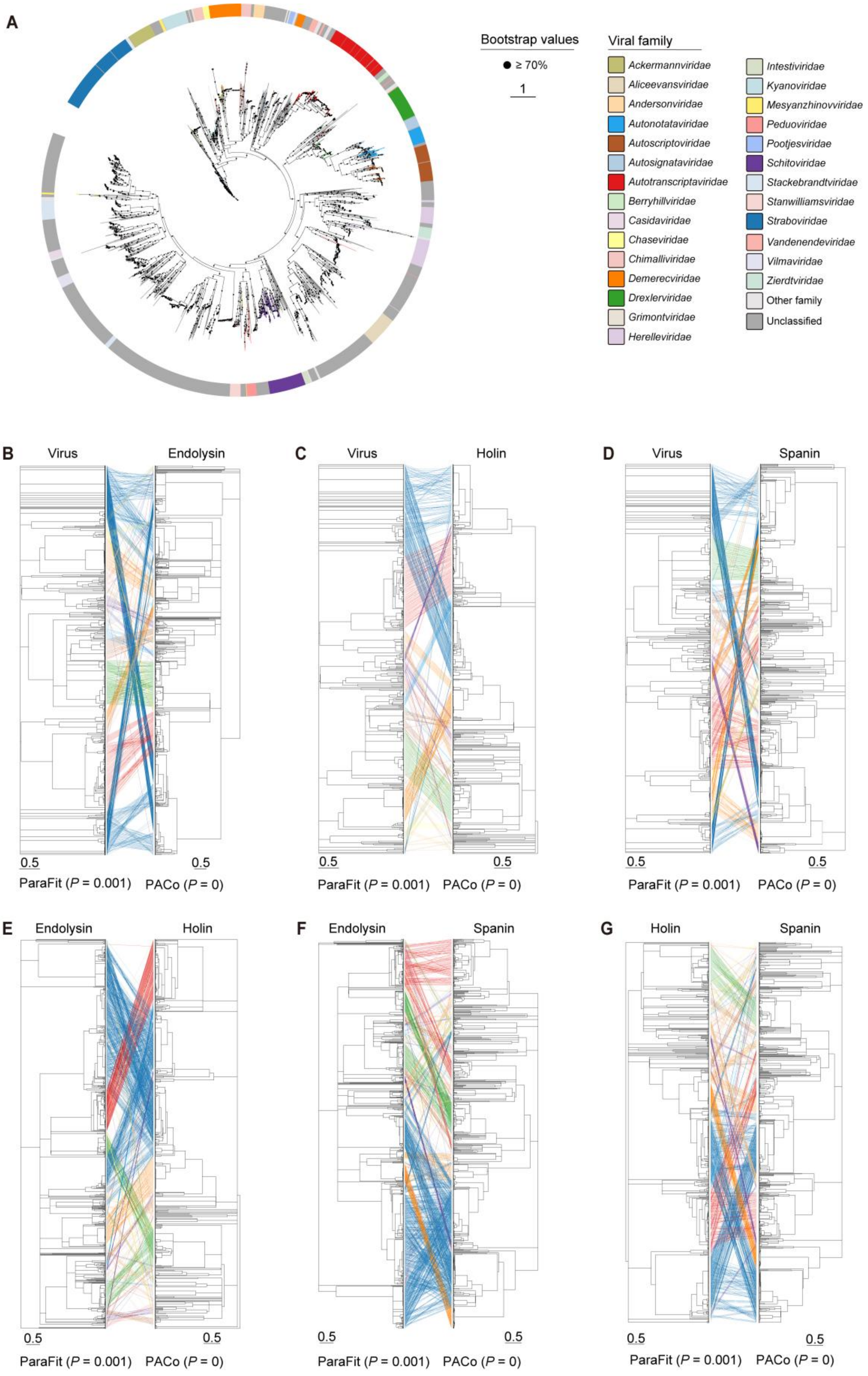

A
Bootstrap values
● ≥ 70%
1
Viral family
Ackermannviridae
Aliceevansviridae
Andersonviridae
Autonotataviridae
Autoscriptoviridae
Autosignataviridae
Autotranscriptaviridae
Berryhillviridae
Casidaviridae
Chaseviridae
Chimalliviridae
Demerecviridae
Drexlerviridae
Grimontviridae
Herelleviridae
Intestiviridae
Kyanoviridae
Mesyanzhinovviridae
Peduoviridae
Pootjesviridae
Schitoviridae
Stackebrandtviridae
Stanwilliamsviridae
Straboviridae
Vandenendeviridae
Vilmaviridae
Zierdtviridae
Other family
Unclassified
B
Virus
Endolysin
0.5
0.5
ParaFit (P = 0.001)
PACo (P = 0)
C
Virus
Holin
0.5
0.5
ParaFit (P = 0.001)
PACo (P = 0)
D
Virus
Spanin
0.5
0.5
ParaFit (P = 0.001)
PACo (P = 0)
E
Endolysin
Holin
0.5
0.5
ParaFit (P = 0.001)
PACo (P = 0)
F
Endolysin
Spanin
0.5
0.5
ParaFit (P = 0.001)
PACo (P = 0)
G
Holin
Spanin
0.5
0.5
ParaFit (P = 0.001)
PACo (P = 0)

Fig. S12. Cophylogenetic analysis of lytic proteins in *Caudoviricetes* viruses. (A) Phylogenetic tree of 2,870 *Caudoviricetes* viruses constructed based on 38 marker proteins. Branches and outer rings are colored according to viral family (ICTV classification) [10]. Scale bar indicates 1 amino acid substitution per site. Black dots at internal nodes indicate bootstrap support values ≥ 70%. **(B-D)** Phylogenetic congruence between *Caudoviricetes* viral species and their LyPs, with connecting lines indicating corresponding branches in the phylogenetic trees. **(E-G)** Phylogenetic congruence among LyPs of *Caudoviricetes* viruses. All phylogenetic congruences were analyzed for co-evolutionary relationships using PACo [11] and ParaFit [12]. Scale bar indicates 0.5 amino acid substitution per site.

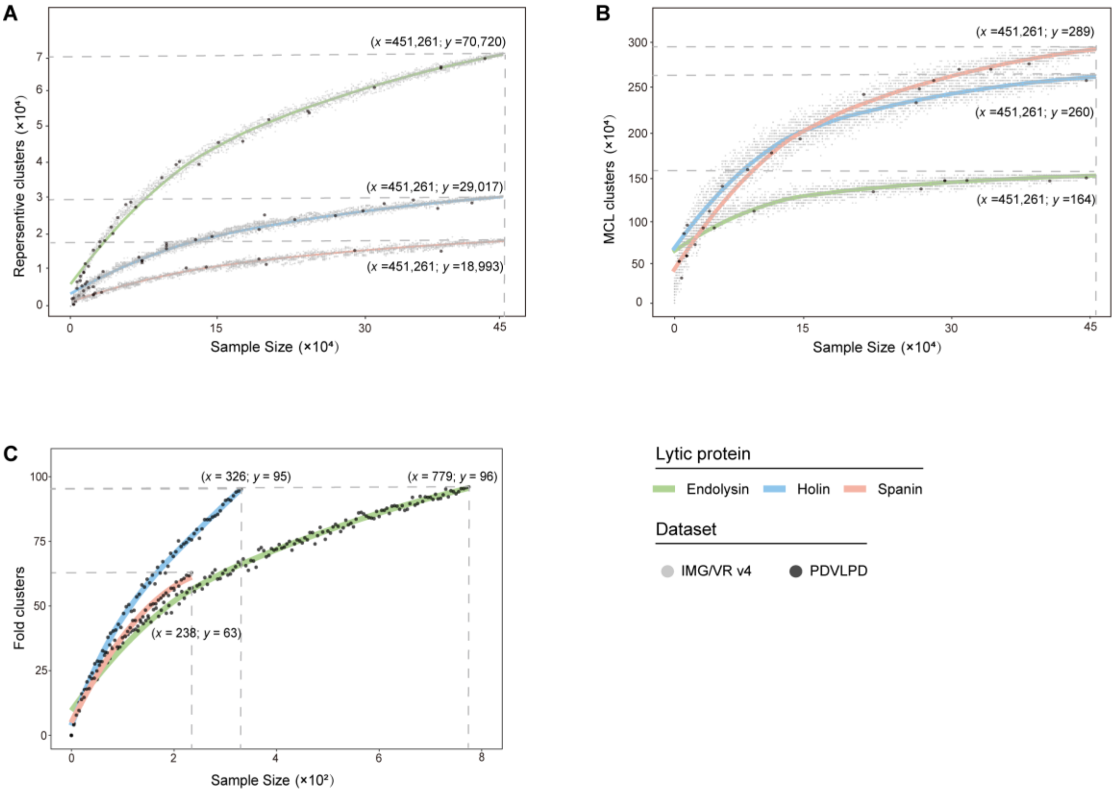

Fig. S13. Saturation analysis of prokaryotic virus lytic protein diversity. (A) Saturation curve of representative LyP sequence clusters. **(B)** Saturation curve of LyP clusters generated via the Markov Cluster Algorithm (MCL) [13] with an inflation parameter (*I*) = 2.0. **(C)** Saturation curve derived from three-dimensional structural clustering of LyPs. Green, blue, and red lines indicate endolysin, holin, and spanin, respectively, showing the best-fit curves from 10 rounds of random sampling for each protein group. Black and gray dots represent protein clusters obtained from PDVLPD and IMG/VR v4 [14] databases, respectively.

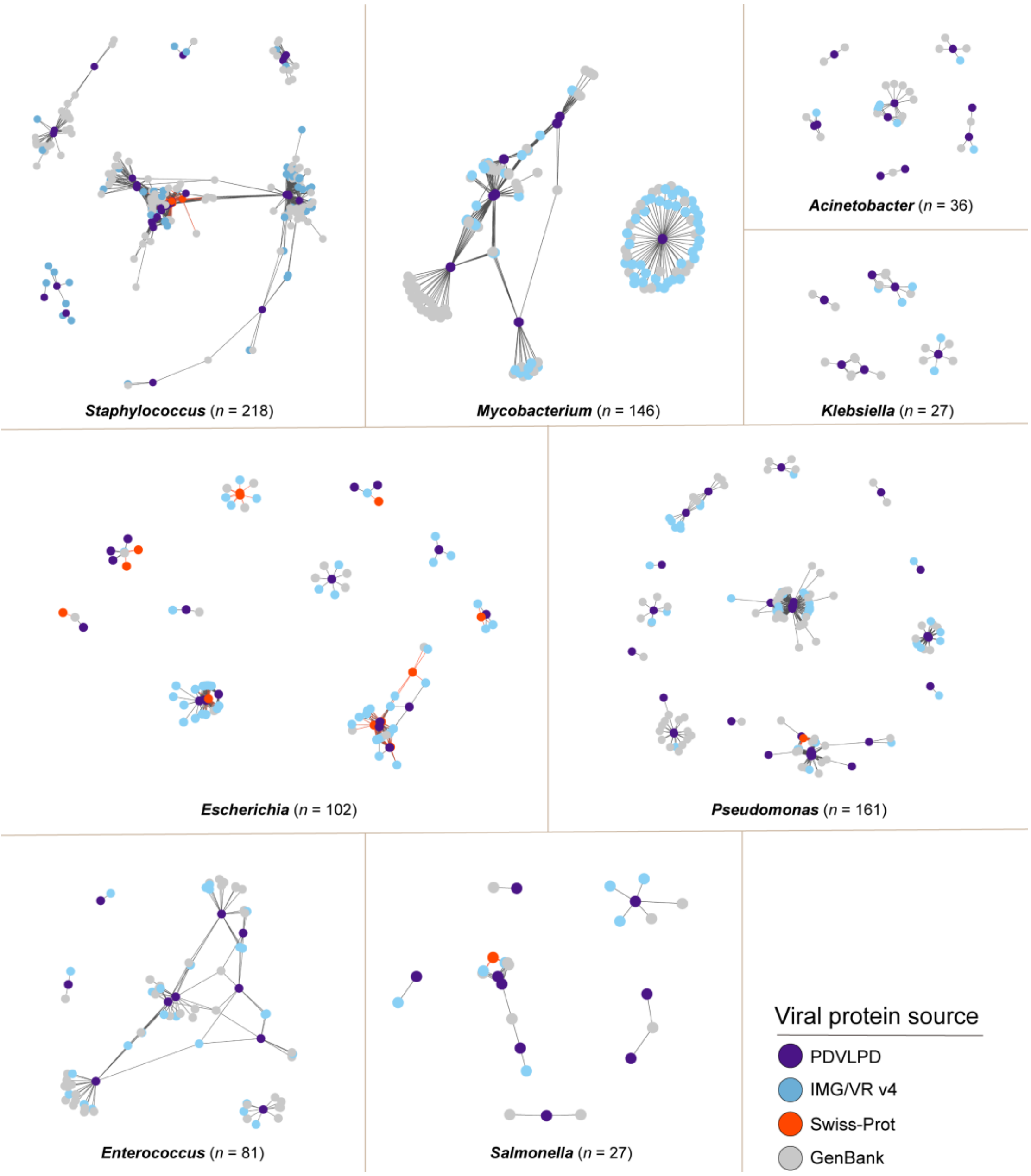

Fig. S14. Similarity-based clustering network of bacteriophage endolysins targeting key pathogenic genera as classified by the WHO. Node colors denote the source database: purple for PDVLPD, blue for IMG/VR v4 [2], and orange for GenBank [15] and Swiss-Prot [16]. Edges between nodes reflect amino acid sequence similarity, with shorter edges indicating higher similarity. The total number of nodes (protein clusters) within each network is indicated in parentheses following the corresponding bacterial genus name.

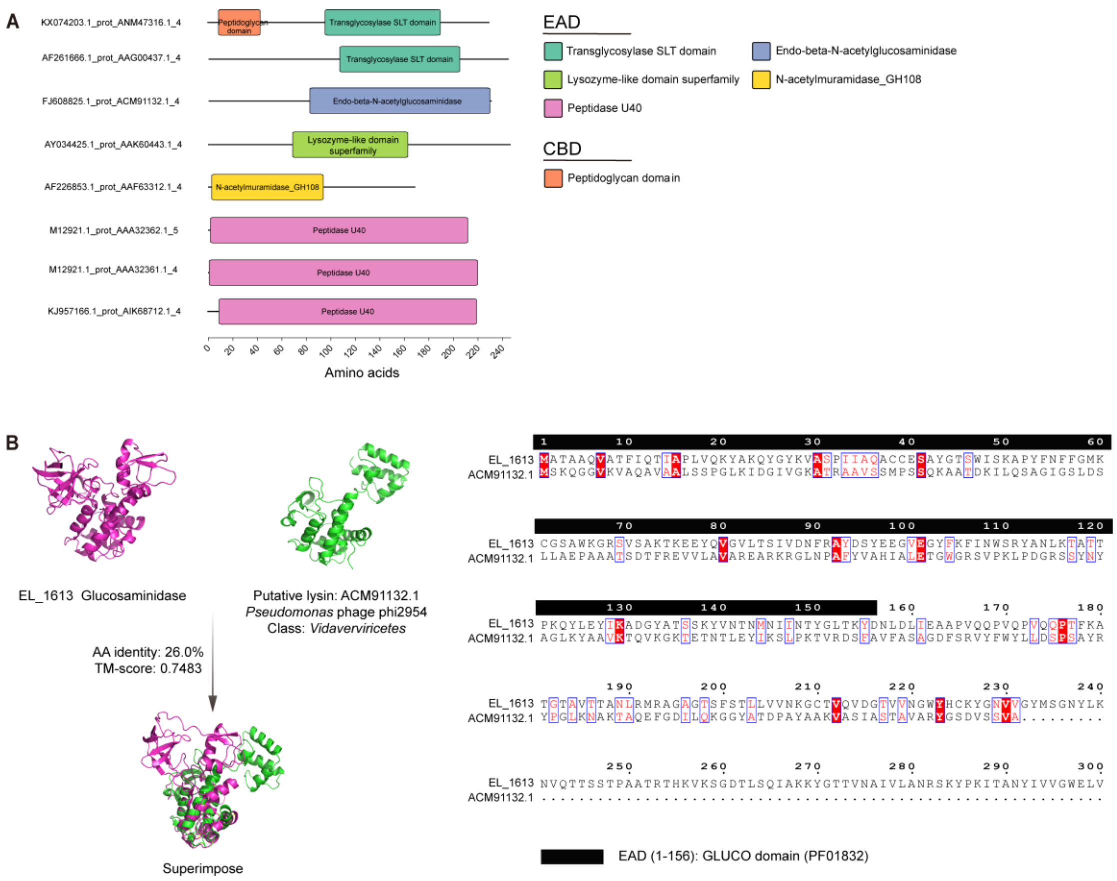

Fig. S15. Domain architecture and tertiary structure analysis of dsRNA virus-encoded endolysins. (A) Schematic representation of conserved domains in 8 dsRNA viral endolysins. EAD: enzymatically active domain; CBD: cell wall binding domain. **(B)** Structural comparison between RNA viral endolysins and their most similar DNA viral endolysin counterparts from the PDVLPD database. Upper and lower panels display comparative results of protein tertiary structures and conserved amino acid sequences, respectively. Identical residues are highlighted in red, and the conserved GLUCO (PF01832) domain is delineated by a black box. TM-scores were calculated using Foldseek (v1.3) [5] easy-search mode.

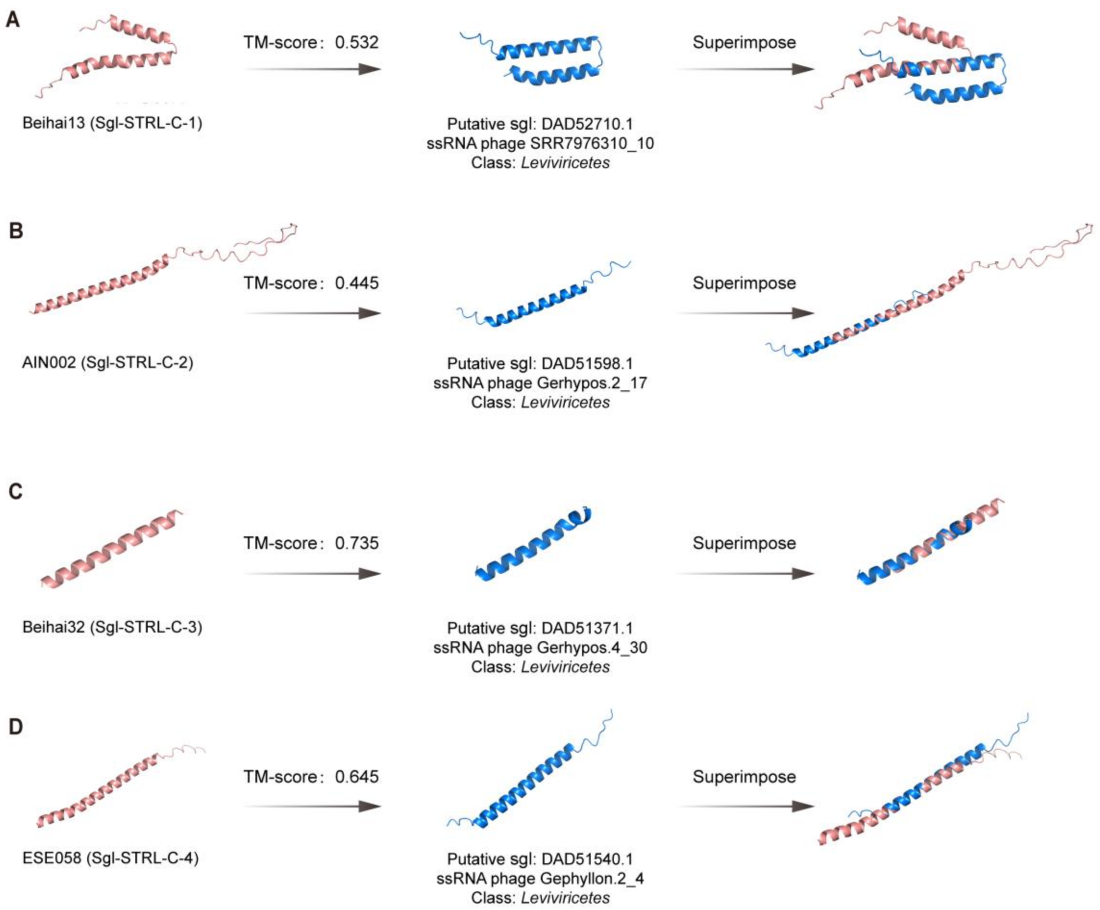

Fig S16. Predicted tertiary structures of ssRNA virus-encoded Sgl proteins. The figure displays structural alignment analyses between four ssRNA virus-encoded Sgl proteins (DAD52710.1, DAD51598.1, DAD51371.1, DAD51540.1) and known functional proteins. These alignments reveal conserved spatial configurations in structural motif regions, suggesting the existence of structural conservation. The clustered Sgl structures were analyzed using TM-align (2021/05/29) [17], with structural similarity evaluated by TM-score.

Supplementary Tables

Table S1. Detailed information of the prokaryotic viral lytic protein query dataset.

Table S2. Detailed information of the prokaryotic DNA virus lytic protein dataset (PDVLPD).

Table S3. Newly identified lytic proteins in prokaryotic viruses based on protein structural similarity.

Table S4. Predicted tertiary structures of ssRNA virus-encoded Sgl proteins.

Table S5. Detailed information on the prokaryotic viral lysis system (PDVLPD).

All Supplementary Tables are available at Figshare (DOI: 10.6084/m9.figshare.28425179)

Supplementary References

1. Finn RD, Clements J, Eddy SR. HMMER web server: interactive sequence similarity searching. *Nucleic Acids Res*. 2011;**39**:W29-37.
2. Zerbini FM, Siddell SG, Lefkowitz EJ *et al.* Changes to virus taxonomy and the ICTV statutes ratified by the international committee on taxonomy of viruses (2023). *Arch Virol*. 2023;**168**:175.
3. Bouras G, Nepal R, Houtak G *et al.* Pharokka: a fast scalable bacteriophage annotation tool. *Bioinformatics*. 2023;**39**:btac776.
4. Cook R, Telatin A, Bouras G *et al.* Driving through stop signs: predicting stop codon reassignment improves functional annotation of bacteriophages. *ISME Commun*. 2024;**4**:ycae079.
5. Barrio-Hernandez I, Yeo J, Jänes J *et al.* Clustering predicted structures at the scale of the known protein universe. *Nature*. 2023;**622**:637-45.
6. Nguyen LT, Schmidt HA, von Haeseler A *et al.* IQ-TREE: a fast and effective stochastic algorithm for estimating maximum-likelihood phylogenies. *Mol Biol Evol*. 2015;**32**:268-74.
7. Xie J, Chen Y, Cai G *et al.* Tree Visualization By One Table (tvBOT): a web application for visualizing, modifying and annotating phylogenetic trees. *Nucleic Acids Res*. 2023;**51**:W587-W92.
8. Shen W, Ren H. TaxonKit: a practical and efficient NCBI taxonomy toolkit. *J Genet Genomics*. 2021;**48**:844-50.
9. Parks DH, Chuvochina M, Rinke C *et al.* GTDB: an ongoing census of bacterial and archaeal diversity through a phylogenetically consistent, rank normalized and complete genome-based taxonomy. *Nucleic Acids Res*. 2022;**50**:D785-D94.
10. Simmonds P, Adriaenssens EM, Lefkowitz EJ *et al.* Changes to virus taxonomy and the ICTV statutes ratified by the international committee on taxonomy of viruses (2024). *Arch Virol*. 2024;**169**:236.
11. Balbuena JA, Míguez-Lozano R, Blasco-Costa I. PACo: a novel procrustes application to cophylogenetic analysis. *PLoS One*. 2013;**8**:e61048.
12. Legendre P, Desdevises Y, Bazin E. A statistical test for host–parasite coevolution. *Syst Biol*. 2002;**51**:217-34.
13. Smyth P. Clustering sequences with hidden markov models. In: *Proceedings of the 10th International Conference on Neural Information Processing Systems, Denver, Colorado*, pp. 648–54: MIT Press, 1996.
14. Camargo AP, Nayfach S, Chen IA *et al.* IMG/VR v4: an expanded database of uncultivated virus genomes within a framework of extensive functional, taxonomic, and ecological metadata. *Nucleic Acids Res*. 2023;**51**:D733-D43.
15. Haft DH, Badretdin A, Coulouris G *et al.* Refseq and the prokaryotic genome annotation pipeline in the age of metagenomes. *Nucleic Acids Res*. 2024;**52**:D762-D69.
16. Boeckmann B, Bairoch A, Apweiler R *et al.* The SWISS-PROT protein knowledgebase and its supplement TrEMBL in 2003. *Nucleic Acids Res*. 2003;**31**:365-70.
17. Zhang Y, Skolnick J. TM-align: a protein structure alignment algorithm based on the TM-score. *Nucleic Acids Res*. 2005;**33**:2302-9.
